# The Fragment HMGA2-sh-3p20 from HMGA2 mRNA 3′UTR Promotes the Growth of Hepatoma Cells by Upregulating HMGA2

**DOI:** 10.1038/s41598-017-02311-0

**Published:** 2017-05-18

**Authors:** Yuan Wang, Fuquan Chen, Zhe Yang, Man Zhao, Shuqin Zhang, Yuen Gao, Jinyan Feng, Guang Yang, Weiying Zhang, Lihong Ye, Xiaodong Zhang

**Affiliations:** 1 State Key Laboratory of Medicinal Chemical Biology, Department of Cancer Research, College of Life Sciences, Nankai University, Tianjin, 300071 China; 2 State Key Laboratory of Medicinal Chemical Biology, Department of Biochemistry, College of Life Sciences, Nankai University, Tianjin, 300071 China

## Abstract

High mobility group A2 (HMGA2) plays a crucial role in the development of cancer. However, the mechanism by which HMGA2 promotes the growth of hepatocellular carcinoma (HCC) remains unclear. Here, we explore the hypothesis that HMGA2 may enhance the growth of hepatoma cells through a fragment based on the secondary structure of HMGA2 mRNA 3′-untranslated region (3′UTR). Bioinformatics analysis showed that HMGA2 mRNA displayed a hairpin structure within its 3′UTR, termed HMGA2-sh. Mechanistically, RNA immunoprecipitation assays showed that the microprocessor Drosha or DGCR8 interacted with HMGA2 mRNA in hepatoma cells. Then, Dicer contributes to the generation of the fragment HMGA2-sh-3p20 from the HMGA2-sh. HMGA2-sh-3p20 was screened by PCR analysis. Interestingly, HMGA2-sh-3p20 increased the expression of HMGA2 through antagonizing the tristetraprolin (TTP)-mediated degradation of HMGA2. HMGA2-sh-3p20 inhibited the expression of PTEN by targeting the 3′UTR of PTEN mRNA. In addition, the overexpression of PTEN could downregulate HMGA2 expression. Significantly, we documented the ability of HMGA2-sh-3p20 to promote the growth of hepatoma cells *in vitro* and *in vivo*. Thus, we conclude that the fragment HMGA2-sh-3p20 from HMGA2 mRNA 3′UTR promotes the growth of hepatoma cells by upregulating HMGA2. Our finding provides new insights into the mechanism by which HMGA2 enhances hepatocarcinogenesis.

## Introduction

High mobility group A2 (HMGA2) is defined as a small nuclear protein with high mobility. HMGA2 can modulate multiple gene expressions through affecting the interactions between protein–DNA or protein–protein^1, 2^. As an oncoprotein, HMGA2 is frequently upregulated in a variety of cancers, such as breast cancer, ovarian cancer, colorectal cancer and lung cancer^3–5^. However, the expression of HMGA2 has not been reported in clinical hepatocellular carcinoma (HCC). Importantly, HMGA2 expression is associated with the serious extent of carcinoma and the clinical outcome^6^. The expression of HMGA2 is regulated by various mechanisms. HMGA2 can be increased by Wnt/β-catenin pathway and decreased by ZBRK1/BRCA1/CtIP pathway. It also can be regulated by microRNAs (miRNAs), such as let7 and miR-182^7–9^. However, the mechanism of HMGA2 regulation in HCC is unclear.

Recently, more and more evidence has showed that mRNAs possess the regulatory properties to modulate gene expression independent of its protein-coding function. It has been reported that the mRNA transcripts can regulate coding gene expression by competing for binding with miRNAs^10^. Besides, accumulated evidence suggests that the mRNA secondary structures especially involving regulatory elements may be closely associated with the regulatory function^11^. Basically, the hairpin is one of the most important structural elements of mRNAs^12^. Recently, our group has reported that a hairpin within the YAP (or PTEN) mRNA 3′-untranslated region (3′UTR) can serve as a regulatory element, which modulates the gene expression through generating a fragment^13, 14^. Therefore, the elements such as hairpin based on the secondary structure of mRNAs play crucial roles in the gene regulation in the cells. Drosha and DGCR8, a part of the nuclear microprocessor complex, are double strands RNA binding proteins and are capable of processing stem-loop structures into short hairpins. Then hairpins generate miRNAs by Dicer, which is essential for the maturation of miRNAs^15, 16^. In addition, Drosha/DGCR8 complex and dicer can directly bind with mRNAs depending on its stem-loop structure and regulate stability or alternative splicing of mRNAs^17–19^. However, whether HMGA2 mRNA has regulatory function is still largely unknown.

In general, more than 100 RNA binding proteins (RBPs) have been identified^20^, while only a small part of RBPs has been well studied. It has been reported that many binding sites of RBPs are located in mRNA 3′UTRs^21, 22^. Tristetraprolin (TTP), a member of RBPs, is a physiological stimulator of the mRNAs instability^23^. With its ability of binding to mRNAs containing AU-rich elements (ARE), TTP can destabilize mRNAs by promoting the removal of mRNAs poly (A) tails or recruiting the RNA decay machinery^24–26^. However, the effect of TTP on HMGA2 mRNA has not been reported. The phosphatase and tensin homolog (PTEN) is an important tumor suppressor. Impaired PTEN function has an effect on genomic instability, DNA repair and cell proliferation^27^. However, the effect of HMGA2 on PTEN is poorly understood.

In the present study, we investigated the mechanism by which HMGA2 enhanced hepatocarcinogenesis based on its secondary structure. Our data showed that the fragment HMGA2-sh-3p20 from HMGA2 mRNA 3′UTR promotes the growth of hepatoma cells by upregulating HMGA2. Our finding provides new insights into the mechanism by which HMGA2 promotes hepatocarcinogenesis.

## Results

### A hairpin within HMGA2 mRNA 3′UTR has regulatory function

**Figure 1:**
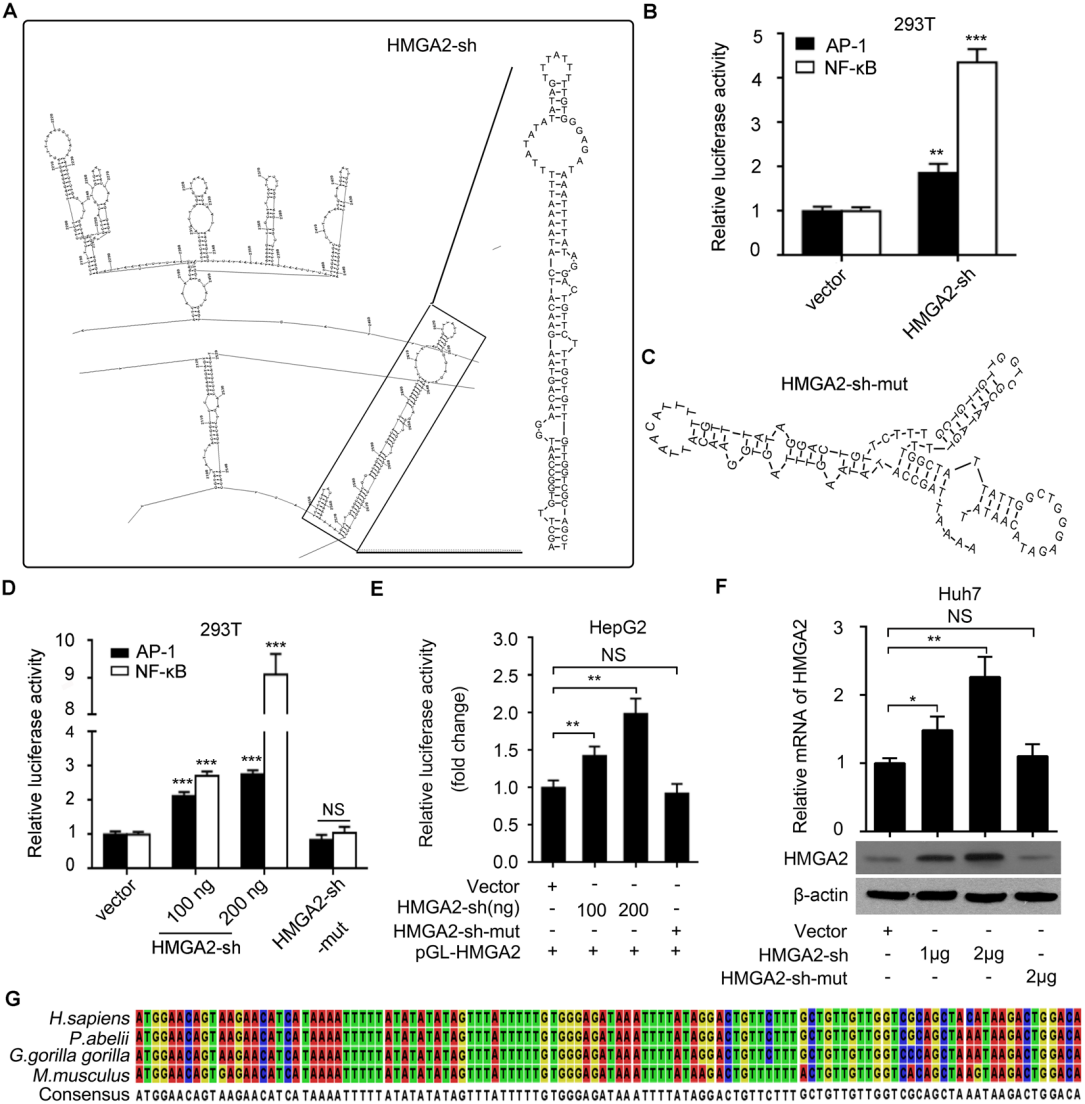
A hairpin within HMGA2 mRNA 3′UTR has regulatory function. (**A**) The secondary structure of HMGA2 3′UTR was analyzed by using RNAstructure. (**B**) The promoter activities of AP-1 and NF-κB were examined by luciferase reporter gene assays in 293T cells. (**C**) The secondary structure of HMGA2-sh-mut with cripple of HMGA2-sh sequence was analyzed by using RNAstructure. (**D**) The promoter activities of AP-1 and NF-κB were measured by luciferase reporter gene assays in 293T cells. (**E**) The luciferase activities of pGL3-HMGA2 were determined by luciferase reporter gene assays in HepG2 cells. (**F**) The expression of HMGA2 was assessed by qRT-PCR and Western blot analysis in Huh7 cells. (**G**) The sequence alignment of HMGA2-sh in different species was analyzed by the software of CLC sequence viewer 6.3. Every experiment was repeated three times. Error bars represent s.d. (n = 3), **p < 0.01; ***p < 0.001 and not significant (NS), Student’s *t* test.

Given that the functions of RNAs are closely associated with their secondary structures, we first predicted the secondary structures of 196 mRNA 3′UTRs by using the software of RNAdraw^28^ and RNAstructure^29^. Our data showed that the structures of mRNA 3′UTRs were complicated at different free energy levels, 10.7% (21/196) mRNA 3′UTRs displayed the hairpins that were relatively stable as compared with other hairpin structures (Supplementary Table S1). Interestingly, the 3′UTR of HMGA2 mRNA contained a stable hairpin structure (termed HMGA2-sh) as well (Supplementary Fig. S1A and Fig. 1A). It has been reported that the transcription factors AP-1 and NF-κB can be affect by a variety of genes related to cell proliferation; they can be markers for cell responses^30^. To address the effect of HMGA2-sh on the cells, we constructed a vector with U6 promoter for the overexpression of HMGA2-sh. Then, we examined the responses of cells to the overexpression of HMGA2-sh by using the luciferase reporter gene system of AP-1 and NF-κB. We observed that HMGA2-sh increased the luciferase activities of AP-1 and NF-κB in 293T cells (Fig. 1B). To explore the effect of HMGA2-sh structure on its activities, we further cloned the mutant of HMGA2-sh (HMGA2-sh-mut) with a cripple sequence of HMGA2-sh, which failed to form hairpin structure (Fig. 1C). We observed that HMGA2-sh increased the luciferase activities of AP-1 and NF-κB in a dose-dependent manner in 293T cells (Fig. 1D), while HMGA2-sh-mut failed to work, suggesting that the structured-HMGA2-sh possesses biological activities. Accordingly, a fragment of 247 bp HMGA2 3′UTR containing HMGA2-sh were constructed into pGL3-control vector, termed pGL3-HMGA2. Indeed, the luciferase reporter gene assays showed that HMGA2-sh could upregulate the luciferase activities of pGL3-HMGA2 in HepG2 (or 293T) cells, rather than HMGA2-sh-mut (Fig. 1E and Supplementary Fig. S1B). Moreover, quantitative real-time PCR (qRT-PCR) and Western blot analysis showed that the overexpression of HMGA2-sh could upregulate the expression of HMGA2 at the levels of mRNA and protein in a dose-dependent manner in hepatoma Huh7 (or HepG2) cells (Fig. 1F and Supplementary Fig. S1C), suggesting that HMGA2-sh as an element has regulatory functions. To better understand the biological significance of the hairpin, we aligned the sequence of HMGA2-sh in different species and found that it was highly conserved in higher mammals (Fig. 1G), suggesting that the hairpin HMGA2-sh may be involved in the event of precise regulation of genes. Thus, we conclude that a hairpin within HMGA2 mRNA 3′UTR has regulatory function.

### A fragment, HMGA2-sh-3p20, is cleaved from HMGA2-sh by Drosha/DGCR8 complex and Dicer

**Figure 2:**
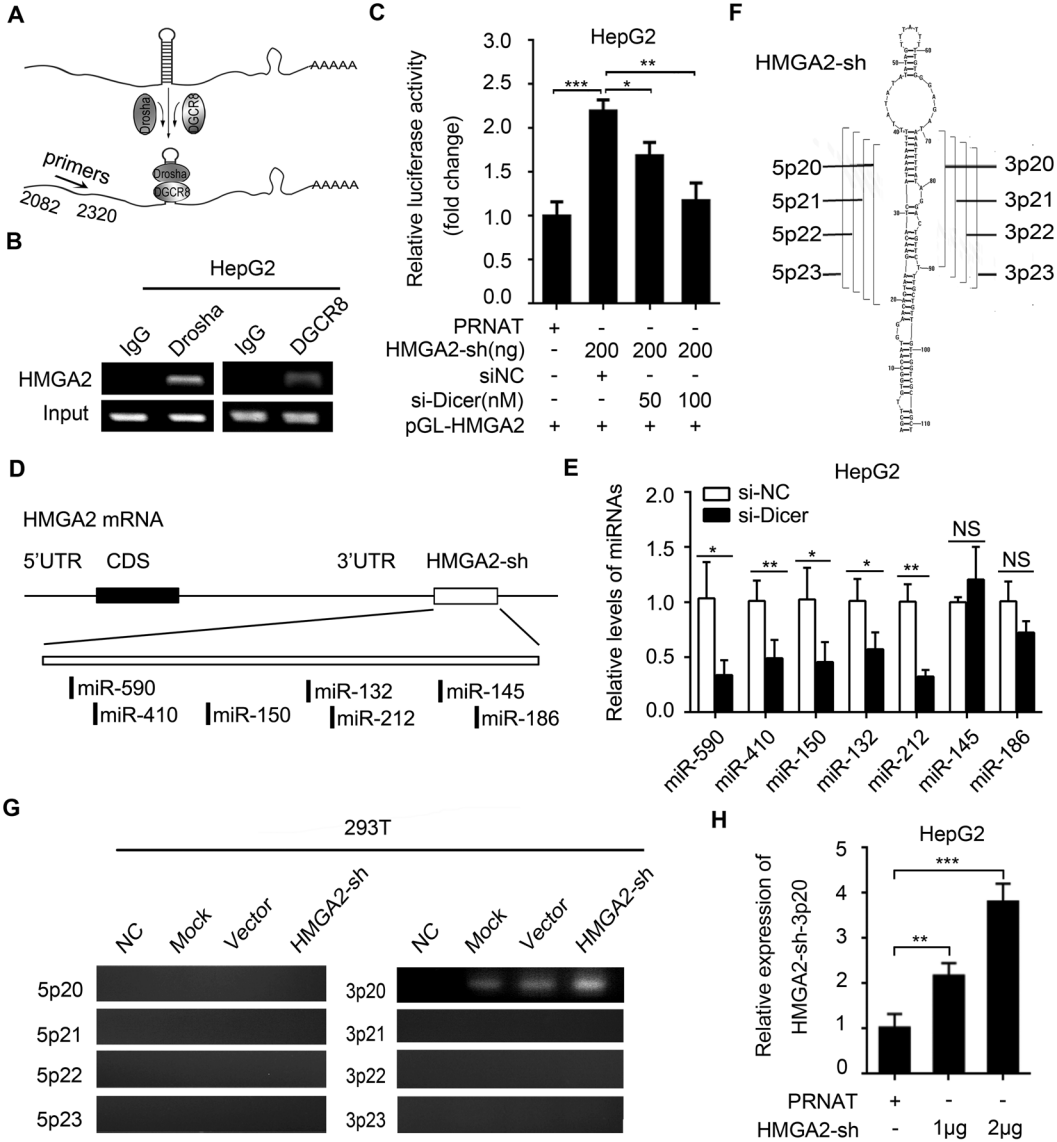
A fragment, HMGA2-sh-3p20, is cleaved from HMGA2-sh by Drosha/DGCR8 complex and Dicer. (**A**) The diagram of the interaction between Drosha/DGCR8 and the hairpin structure. (**B**) Drosha (or DGCR8) RIP-PCR of HMGA2 mRNA in HepG2 cells. (**C**) Effect of si-Dicer on luciferase activities of HMGA2-sh-mediated pGL3-HMGA2 was measured by luciferase reporter gene assays in HepG2 cells. (**D**) The diagram shows the predicted miRNAs targeting pGL-HMGA2. (**E**) Effect of si-Dicer on the expression of miR-590, miR-410, miR-150, miR-132/212, miR-145 and miR-186 was examined by qRT-PCR in HepG2 cells. (**F**) The diagram shows the designed primers for fragments derived from HMGA2-sh. (**G**) The fragments were screened by RT-PCR using designed primers in HMGA2-sh-overexpressed 293T cells. NC, purified water; Mock, without plasmid DNA; Vector presents empty plasmid DNA. The full bands images are given as Supplementary Fig. S6. (**H**) The identified fragment, HMGA2-sh-20, was validated by qRT-PCR analysis in HepG2 cells transiently transfected with HMGA2-sh. Every experiment was repeated three times. Error bars represent s.d. (n = 3), **p < 0.01; ***p < 0.001, Student’s *t* test.

Given that the primary miRNA transcripts (pri-miRNAs) can be processed into miRNAs by Drosha/DGCR8 and Dicer^31^, we verified whether HMGA2-sh could generate a fragment through Drosha/DGCR8 complex and Dicer as well (Fig. 2A). Interestingly, RNA immunoprecipitation (RIP) assays showed that HMGA2 mRNA could be immunoprecipitated by Drosha or DGCR8 in HepG2 cells (Fig. 2B), suggesting that Drosha/DGCR8 complex is responsible for the process of HMGA2-sh. Moreover, HMGA2-sh-mediated upregulation of pGL-HMGA2 luciferase activities could be disrupted by si-Dicer in HepG2 cells (Fig. 2C), in which the efficiency of si-Dicer was validated (Supplementary Fig. S2A), suggesting that Dicer contributes to the cleavage of HMGA2-sh in the cells. Since Dicer can affect the expression of miRNAs, we predicted some miRNAs (miR-590, miR-410, miR-150, miR-132/212, miR-145 and miR-186) targeting pGL-HMGA2 (Fig. 2D) and examined the effect of si-Dicer on the expression of these miRNAs. Our data showed that the expression of miR-145 and miR-186 were not changed, while the expression of miR-590, miR-410, miR-150 and miR-132/212 were downregulated when Dicer was knocked down in HepG2 cells (Fig. 2E), supporting that the luciferase activities of pGL-HMGA2 is affected by HMGA2-sh, rather than miRNAs. Meanwhile, we revealed that miR-132 and miR-150 failed to affect the level of HMGA2 mRNA (Supplementary Fig. S2B and C). Next, we supposed that a fragment might be cleaved from HMGA2-sh in the cells. Accordingly, we designed the different PCR primers for HMGA2-sh (Fig. 2F). Interestingly, PCR analysis showed that HMGA2-sh-3p20 was detectable in 293T cells transfected with HMGA2-sh (Fig. 2G). PCR products of HMGA2-sh-3p20 from 293T cells were cloned into pEASY-T1 vector and the constructs were proved by sequencing (Supplementary Fig. S2D). QRT-PCR analysis further validated that the overexpression of HMGA2-sh resulted in the increase of HMGA2-sh-3p20 in a dose-dependent manner in HepG2 cells (Fig. 2H), suggesting that HMGA2-sh-3p20, a fragment, might be cleaved from HMGA2-sh. Taken together, we conclude that a fragment, HMGA2-sh-3p20, is cleaved from HMGA2-sh by Drosha/DGCR8 complex and Dicer.

### HMGA2-sh-3p20 upregulates HMGA2 by blocking the TTP-mediated degradation of HMGA2 mRNA

**Figure 3:**
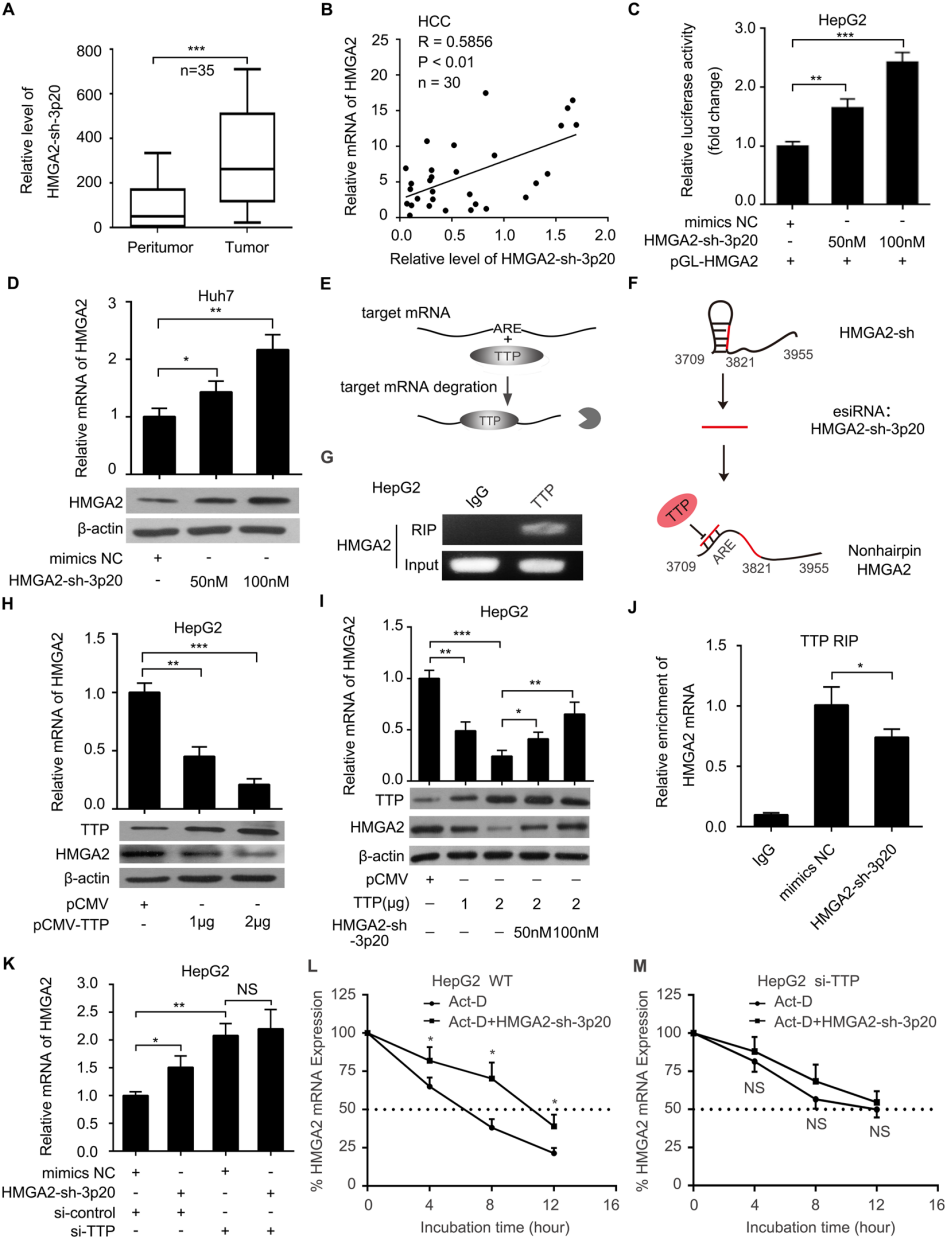
HMGA2-sh-3p20 upregulates HMGA2 by blocking the TTP-mediated degradation of HMGA2 mRNA. (**A**) The relative expression of HMGA2-sh-3p20 was assessed by qRT-PCR in 35 pairs of clinical HCC tissues and corresponding peritumor tissues (***P < 0.001; Wilcoxon’s signed-rank test). (**B**) The correlation between HMGA2 mRNA levels and HMGA2-sh-3p20 levels was measured by qRT-PCR in 30 cases of clinical HCC tissues (**P < 0.01, r = 0.586; Pearson’s correlation coefficient). (**C**) The luciferase activities of pGL3-HMGA2 were examined by luciferase reporter gene assays in HepG2 cells. (**D**) The expression of HMGA2 was assessed by qRT-PCR and Western blot analysis in Huh7 cells. (**E**) The diagram of TTP-mediated mRNA degradation. (**F**) The diagram of HMGA2-sh-3p20 antagonizes the interaction of TTP with non-hairpin within 3′UTR of HMGA2 mRNA. (**G**) TTP RIP-PCR of HMGA2 in HepG2 cells. (**H**) Effect of TTP on the expression of HMGA2 was measured by qRT-PCR and Western blot analysis in HepG2 cells. (**I**) Effect of HMGA2-sh-3p20 on the expression of TTP-mediated HMGA2 was assessed by qRT-PCR and Western blot analysis in HepG2 cells. The full length blots images are given as Supplementary Fig. S7. (**J**) TTP RIP-qPCR of HMGA2 in HepG2 cells transfected with HMGA2-sh-3p20. (**K**) Effect of HMGA2-sh-3p20 on the levels of HMGA2 mRNA in HepG2 cells transfected with si-TTP by qRT-PCR. (**L**,**M**) Effect of HMGA2-sh-3p20 on the half-life of HMGA2 mRNA in HepG2 cells (**L**) or HepG2 cells transfected with si-TTP (**M**) by qRT-PCR. Every experiment was repeated three times. Error bars represent s.d. (n = 3), **p < 0.01; ***p < 0.001 and not significant (NS), Student’s *t* test.

To better understand the significance of HMGA2-sh-3p20, we evaluated its expression in clinical HCC tissues. Interestingly, qRT-PCR assays showed that the levels of HMGA2-sh-3p20 were higher in HCC tissues relative to their peritumor liver tissues in 35 paired clinical HCC samples (*P* < 0.001; Wilcoxon’s signed-rank test, Fig. 3A). Furthermore, the levels of HMGA2-sh-3p20 were positively associated with those of HMGA2 mRNA in the afore-mentioned clinical samples (*P* < 0.01; Pearson correlation, Fig. 3B). Luciferase reporter gene assays showed that HMGA2-sh-3p20 was able to elevate the activities of pGL-HMGA2 containing HMGA2-sh in HepG2 (or 293T) cells (Fig. 3C and Supplementary Fig. S3A). Moreover, the overexpression of HMGA2-sh-3p20 resulted in the upregulation of HMGA2 at the levels of mRNA and protein in a dose-dependent manner in Huh7 (or HepG2) cells (Fig. 3D and Supplementary Fig. S3B). The inhibitor of HMGA2-sh-3p20 failed to downregulate the expression of HMGA2 at the levels of mRNA and protein (Supplementary Fig. S3C), implying that the levels of HMGA2-sh-3p20 are lower in hepatoma cells. However, HMGA2-sh-3p20 inhibitor could decrease the expression of HMGA2 at mRNA levels in a dose-dependent manner in HepG2 cells when transiently transfected with HMGA2-sh (Supplementary Fig. S3D).

It has been reported that TTP can lead to the degradation of mRNA by directly binding to ARE located in the mRNA 3′UTR^32^ (Fig. 3E). MiR-466l upregulates IL-10 mRNA levels by preventing TTP binding to the AREs, resulting in the blockage of TTP-mediated degradation of IL-10 mRNA^33^. Because HMGA2-sh is an AU-rich element, we supposed that HMGA2-sh-3p20 might upregulate HMGA2 through blocking the role of TTP, if HMGA2 mRNA was regulated by TTP (Fig. 3F). Interestingly, RIP assays showed that HMGA2 mRNA could be immunoprecipitated by TTP in HepG2 cells (Fig. 3G). Moreover, overexpression of TTP led to the downregulation of HMGA2 at the levels of mRNA and protein in HepG2 (or Huh7) cells in a dose-dependent manner (Fig. 3H and Supplementary Fig. S3F), suggesting that TTP can modulate the degradation of HMGA2 mRNA through interacting with the HMGA2 mRNA. The transfection efficiency of TTP was validated by qRT-PCR in the cells (Supplementary Fig. S3E). Surprisingly, the overexpression of HMGA2-sh-3p20 was able to rescue the TTP-mediated downregulation of HMGA2 at the levels of mRNA and protein in HepG2 (or Huh7) cells (Fig. 3I and Supplementary Fig. S3I), suggesting that HMGA2-sh-3p20 can compete with TTP for the interaction with its complementary fragment of non-hairpin HMGA2 mRNA 3′UTR, resulting in the elevation of HMGA2 expression. The transfection efficiency of TTP was validated in the cells (Supplementary Fig. S3G and H). Furthermore, RIP assays showed that the overexpression of HMGA2-sh-3p20 could decrease the interaction of HMGA2 mRNA with TTP in HepG2 cells (Fig. 3J). In addition, HMGA2-sh-3p20 failed to upregulate the expression of HMGA2 when HepG2 cells transfected with si-TTP (Fig. 3K). The interference efficiency of TTP was validated by Western blot analysis (Supplementary Fig. S3J). Given that TTP can lead to the degradation of mRNA, the actinomycin D (Act-D), a RNA polymerase inhibitor, was applied to assess the stability of HMGA2 mRNA. QRT-PCR revealed that the transfection of HMGA2-sh-3p20 could extend the half-time of HMGA2 mRNA, but it failed to work when silencing TTP in HepG2 cells (Fig. 3L and M). Thus, we conclude that HMGA2-sh-3p20 antagonizes TTP-mediated the degradation of HMGA2 mRNA by targeting itself HMGA2 mRNA, resulting in upregulation of HMGA2 in hepatoma cells.

### HMGA2-sh-3p20 inhibits PTEN by targeting the 3′UTR of PTEN mRNA

**Figure 4:**
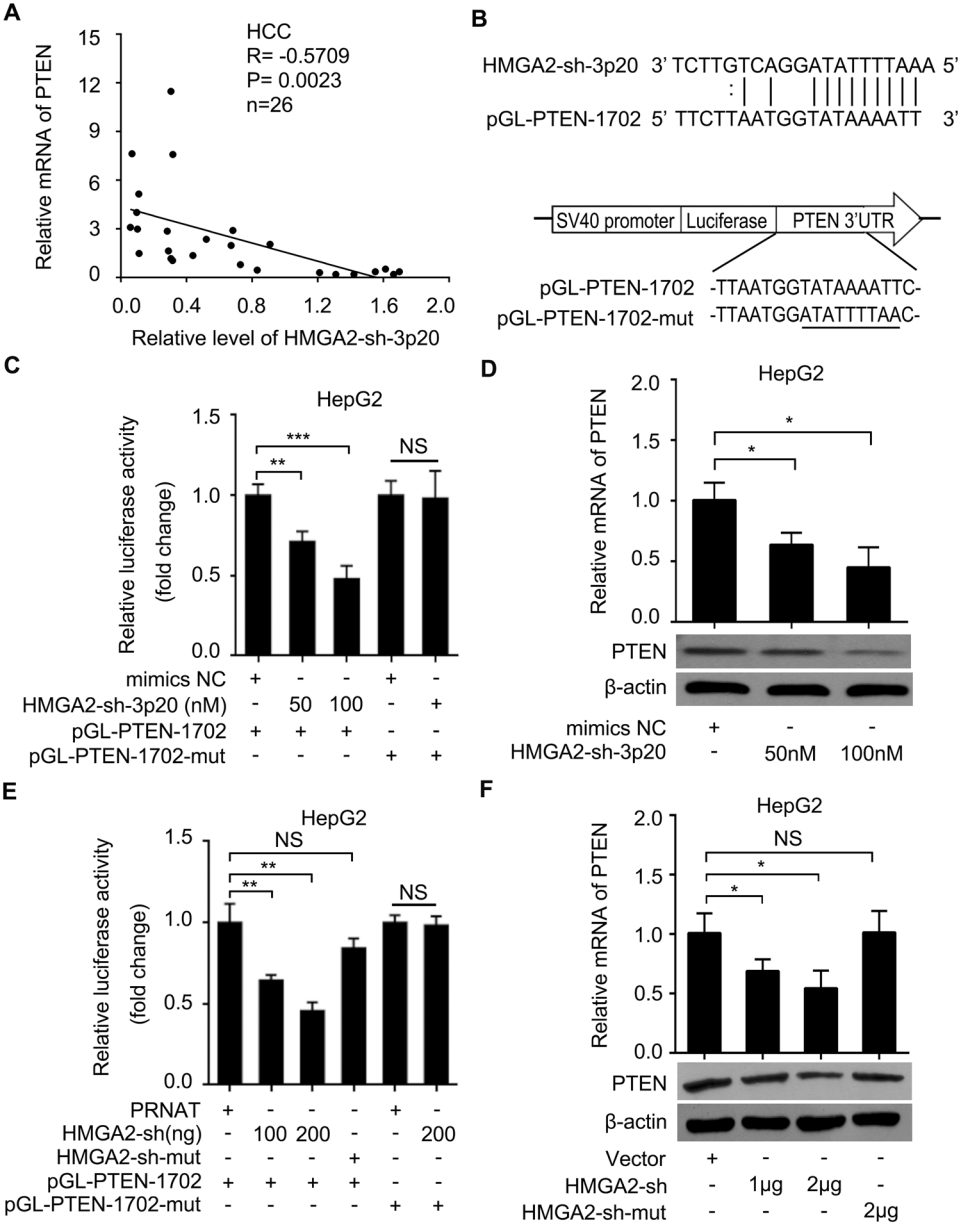
HMGA2-sh-3p20 inhibits PTEN by targeting the 3′UTR of PTEN mRNA. (**A**) The correlation between the levels of PTEN mRNA and HMGA2-sh-3p20 was examined by qRT-PCR in 26 cases of clinical HCC tissues (**P < 0.01, r = −0.571; Pearson’s correlation coefficient). (**B**) A model of predicted HMGA2-sh-3p20 binding site at nucleotides 1702-1722 of the PTEN 3′UTR (pGL3-PTEN-1702) and the mutant of pGL3-PTEN-1702. (**C**) Effect of HMGA2-sh-3p20 on the luciferase activities of pGL3-PTEN-1702 and pGL3-PTEN-1702-mut were measured by luciferase reporter gene assays in HepG2 cells. (**D**) Effect of HMGA2-sh-3p20 on the expression of PTEN was assessed by qRT-PCR and Western blot analysis in HepG2 cells. (**E**) Effect of HMGA2-sh on the luciferase activities of pGL3-PTEN-1702 and pGL3-PTEN-1702-mut were examined by luciferase reporter gene assays in HepG2 cells. (**F**) Effect of HMGA2-sh on the expression of PTEN was measured by qRT-PCR and Western blot analysis in HepG2 cells. Every experiment was repeated three times. Error bars represent s.d. (n = 3), **p < 0.01; ***p < 0.001 and not significant (NS), Student’s *t* test.

Next, we concerned whether HMGA2-sh-3p20 as a fragment could target other mRNAs except targeting itself HMGA2 mRNA. Interestingly, we observed that PTEN mRNA was one of the potential targets of HMGA2-sh-3p20 by using RNAhybrid software^34, 35^. Moreover, qRT-PCR assays showed that the levels of HMGA2-sh-3p20 were negatively associated with those of PTEN in the afore-mentioned clinical samples (*P* < 0.01; Pearson correlation, Fig. 4A). Then, two fragments with HMGA2-sh-3p20 binding site in the 3′UTR of PTEN mRNA were constructed into the vector of pGL3-control (termed pGL-PTEN-1702 and pGL-PTEN-3062), respectively (Fig. 4B). Luciferase reporter gene assays screened that HMGA2-sh-3p20 could reduce the luciferase activities of pGL-PTEN-1702 in 293T cells, rather than those of pGL-PTEN-3062 (Supplementary Fig. S4A). Moreover, we validated that HMGA2-sh-3p20 could decrease the luciferase activities of pGL-PTEN-1702 in a dose-dependent manner in HepG2 (or 293T) cells, but the mutant of pGL-PTEN-1702 (pGL-PTEN-1702-mut) failed to work (Fig. 4C and Supplementary Fig. S4B), suggesting that HMGA2-sh-3p20 is able to target the 3′UTR of PTEN mRNA. Meanwhile, the similar effect of HMGA2-sh on pGL-PTEN-1702 or pGL-PTEN-1702-mut was observed in HepG2 (or 293T) cells (Fig. 4E and Supplementary Fig. S4F). Furthermore, HMGA2-sh-3p20 overexpression suppressed the expression of PTEN at the levels of mRNA and protein in a dose-dependent manner in HepG2 (or Huh7 or LO2) cells (Fig. 4D, Supplementary Fig. S4C and D), and similar effect of HMGA2-sh on PTEN was observed at the levels of mRNA and protein in HepG2 (or Huh7) cells (Fig. 4F and Supplementary Fig. S4G). In addition, qRT-PCR assays showed that HMGA2-sh-3p20 inhibitor increased PTEN expression at the levels of mRNA in a dose-dependent manner in HepG2 cells when transiently transfected with HMGA2-sh (Supplementary Fig. S4E). Next, we examined the effect of PTEN on HMGA2. Interestingly, we observed that the overexpression of PTEN inhibited the expression of HMGA2 at mRNA and protein levels in HepG2 cells. The transfection efficiency of PTEN was validated by qRT-PCR and Western blot analysis (Supplementary Fig. S4H and I). Thus, we conclude that HMGA2-sh-3p20 suppresses the expression of PTEN by targeting the 3′UTR of PTEN mRNA.

### HMGA2-sh-3p20 contributes to the growth of hepatoma cells *in vitro* and *in vivo*

**Figure 5:**
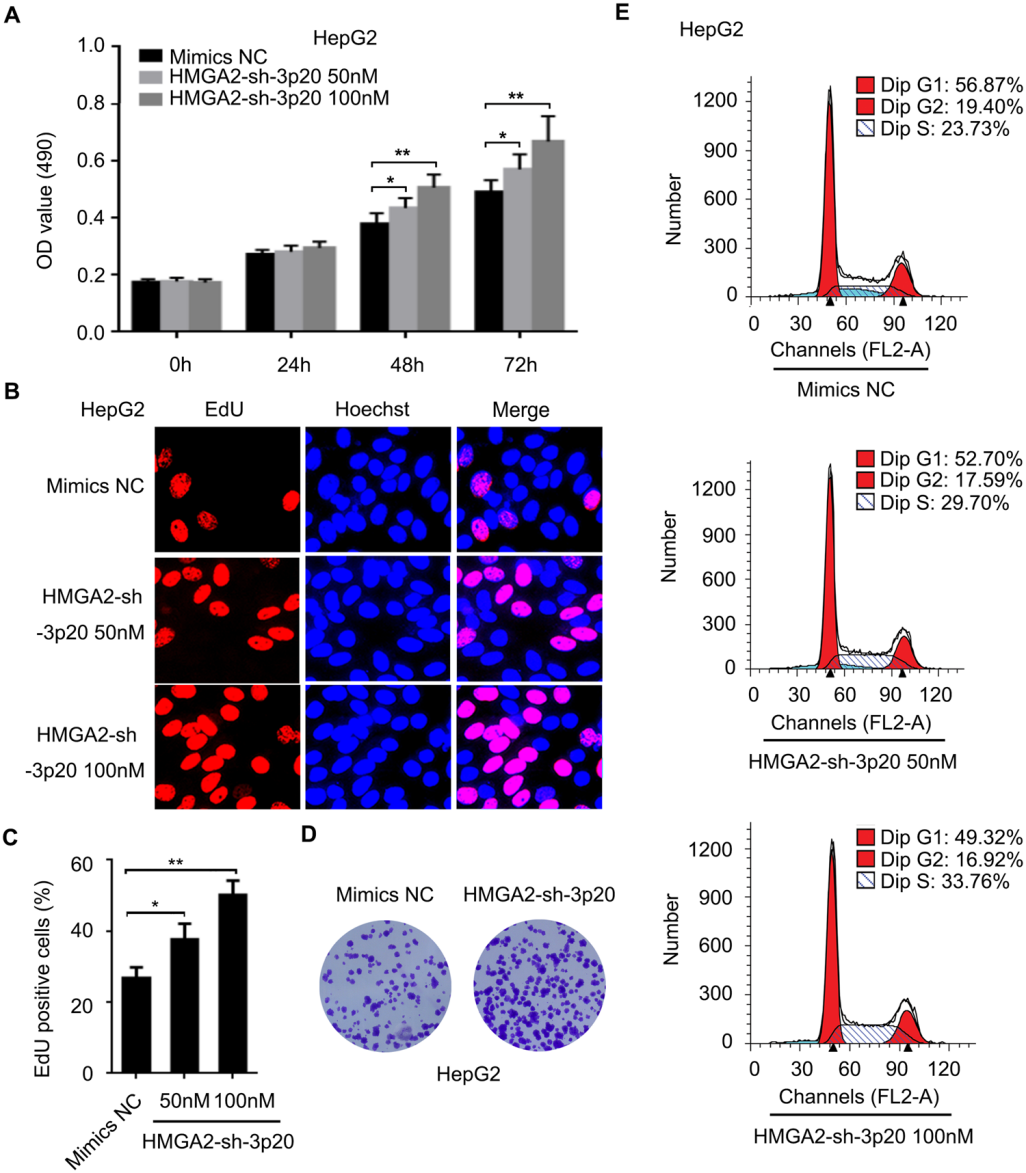
HMGA2-sh-3p20 contributes to the proliferation of hepatoma cells *in vitro*. (**A**) Effect of HMGA2-sh-3p20 on proliferation of HepG2 cells was determined by MTT assays. (**B**,**C**) Effect of HMGA2-sh-3p20 on proliferation of HepG2 cells was measured by EdU incorporation assays. (**D**) Effect of HMGA2-sh-3p20 on proliferation of HepG2 cells was assessed by colony-formation assays. (**E**) Effect of HMGA2-sh-3p20 on proliferation of HepG2 cells was tested by flow cytometry assays. Every experiment was repeated three times. Error bars represent s.d. (n = 3), **p < 0.01; ***p < 0.001 and not significant (NS), Student’s *t* test.

**Figure 6:**
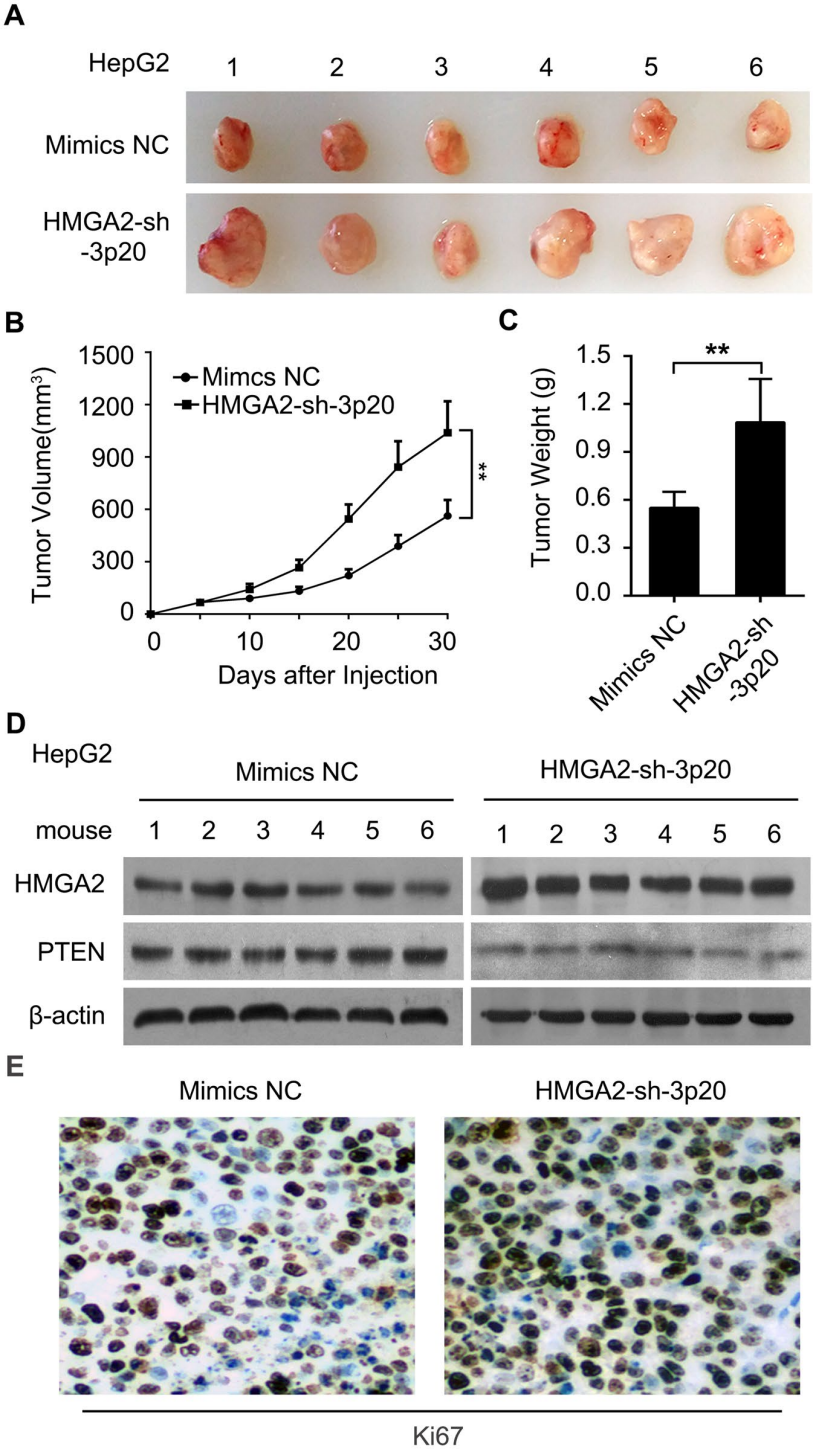
HMGA2-sh-3p20 contributes to the growth of hepatoma cells *in vivo*. (**A**) Photographs of dissected tumors from nude mice tumor transplanted with HepG2 cells pretreated with HMGA2-sh-3p20 or Mimics. (**B**) Growth curve of tumors from experimental groups of nude mice. (**C**) The average weight of tumors from experimental groups of nude mice. (**D**) Protein expression levels of HMGA2 and PTEN were examined by Western blot analysis in the tumor tissues from mice. (**E**) The expression levels of Ki67 were detected by IHC staining in the tumor tissues from mice.

To better understand the significance of HMGA2-sh-3p20 in hepatoma cells, we examined the effect of HMGA2-sh-3p20 on proliferation of hepatoma cells by MTT, EdU, colony-formation and flow cytometry assays in HepG2 (or Huh7) cells. Our data showed that the overexpression of HMGA2-sh-3p20 elevated the proliferation ability of HepG2 (or Huh7) cells (Fig. 5A–E and Supplementary Fig. S5A–E). Then, we subcutaneously injected HepG2 cells that pretreated with the overexpression of HMGA2-sh-3p20 into 4-week-old BALB/c athymic nude mice. Strikingly, we observed that the treatment with HMGA2-sh-3p20 enhanced the growth of HepG2 cells *in vivo* (Fig. 6A–C). Meanwhile, we validated that the expression levels of HMGA2 (or PTEN) were increased (or decreased) in the tumor tissues from mice (Fig. 6D). Accordingly, immunohistochemistry staining (IHC) showed that the levels of Ki67, a cell proliferation marker, were consistent with the tumor volumes as well (Fig. 6E). Therefore, we conclude that HMGA2-sh-3p20 contributes to the growth of hepatoma cells *in vitro* and *in vivo*. Therefore, we conclude that HMGA2-sh-3p20 contributes to the growth of hepatoma cells.

## Discussion

HMGA2 is an architectural transcription factor that regulates the expression of numerous genes^3^. It has been reported that the elements based on secondary structure of mRNAs play crucial roles in gene regulation. In the present study, we investigated the mechanism by which HMGA2 promotes the development of HCC based on its secondary structure.

To better understand the roles of elements based on secondary structure in mRNA regulation, we examined the significance of the secondary structures of 196 mRNA 3′UTRs. Interestingly, our findings showed that around 10% mRNA 3′UTRs contained stable hairpin structures. Notably, we observed a hairpin (HMGA2-sh) within 3′UTR of HMGA2 mRNA. Then, we focused on the investigation that the function of HMGA2-sh in hepatoma cells. Basically, we found that HMGA2-sh was able to generate a fragment, HMGA2-sh-3p20, through Drosha/DGCR8 complex and Dicer, which was consistent with our previous reports^13, 14^. It suggests that the standard hairpins, which can release fragments, within mRNA 3′UTRs are functional elements. Interestingly, we observed that the HMGA2-sh sequences were highly conserved in higher mammals. It implies that HMGA2-sh may be involved in the precise regulation of genes.

Next, we identified the mechanism by which HMGA2-sh-3p20 upregulated the expression of HMGA2 in hepatoma cells. Numerous studies have noted that RBPs and miRNAs are key regulators in the post-transcriptional regulation of genes. It has been reported that TTP can bind to ARE-containing transcripts and destabilizes them by promoting the removal of poly (A) tails. However, the effect of TTP on HMGA2 mRNA is not well documented. Thus, we supposed that TTP might be involved in the regulation of HMGA2 mRNA. Strikingly, we observed that TTP was capable of interacting with HMGA2 mRNA, resulting in the decrease of HMGA2 in hepatoma cells. It suggests that TTP contributes to the downregulation of HMGA2 through interaction with HMGA2 mRNA, which is consistent with that TTP destabilizes mRNAs^36^. It has been reported that MiR-466l could upregulate IL-10 mRNA levels by preventing TTP binding to the IL-10 mRNA^33^. Then, we are interested in whether HMGA2-sh-3p20 increases the levels of HMGA2 mRNA through TTP as well. Strikingly, we show that HMGA2-sh-3p20 upregulates the expression of HMGA2 through antagonizing TTP-mediated HMGA2 degradation in hepatoma cells. Then, we speculated that hairpin and non-hairpin HMGA2 might co-exist in hepatoma cells. It suggests that the formation of hairpin within HMGA2 may depend on the energy of base-pairing in hairpin. The fragment HMGA2-sh-3p20 is cleaved from hairpin HMGA2 mRNA by Drosha/DGCR8 complex, which competes with TTP in non-hairpin HMGA2 mRNA to upregulate HMGA2 at post-transcriptional level. In addition, we identified that PTEN mRNA is another target of HMGA2-sh-3p20. It has been reported that miR-26a is able to inhibit the expression of HMGA2 in gallbladder cancer and non-small cell lung cancer, and upregulate PTEN expression in gastric cancer^37–39^. Our finding is consistent with the reports. However, the binding site of miR-26a in HMGA2 3′UTR (position 1692-1713) is different from that of HMGA2-sh-3p20 (position 3779-3799) in our system. It implies that miR-26a may be involved in upregulation of HMGA2 and downregulation of PTEN besides HMGA2-sh-3p20 in HCC. The other targets of HMGA2-sh-3p20 will be further investigated. In addition, we found that the overexpression of PTEN could downregulate HMGA2 at the levels of mRNA and protein. It suggests that PTEN is able to depress the expression HMGA2 in HCC cells. However, the underlying mechanism is not well documented, which may be related to modulating the methylation of HMGA2 promoter or microRNAs. Our finding provides new insights into the mechanism by which HMGA2 enhances hepatocarcinogenesis based on the secondary structure of HMGA2 mRNA.

**Figure 7:**
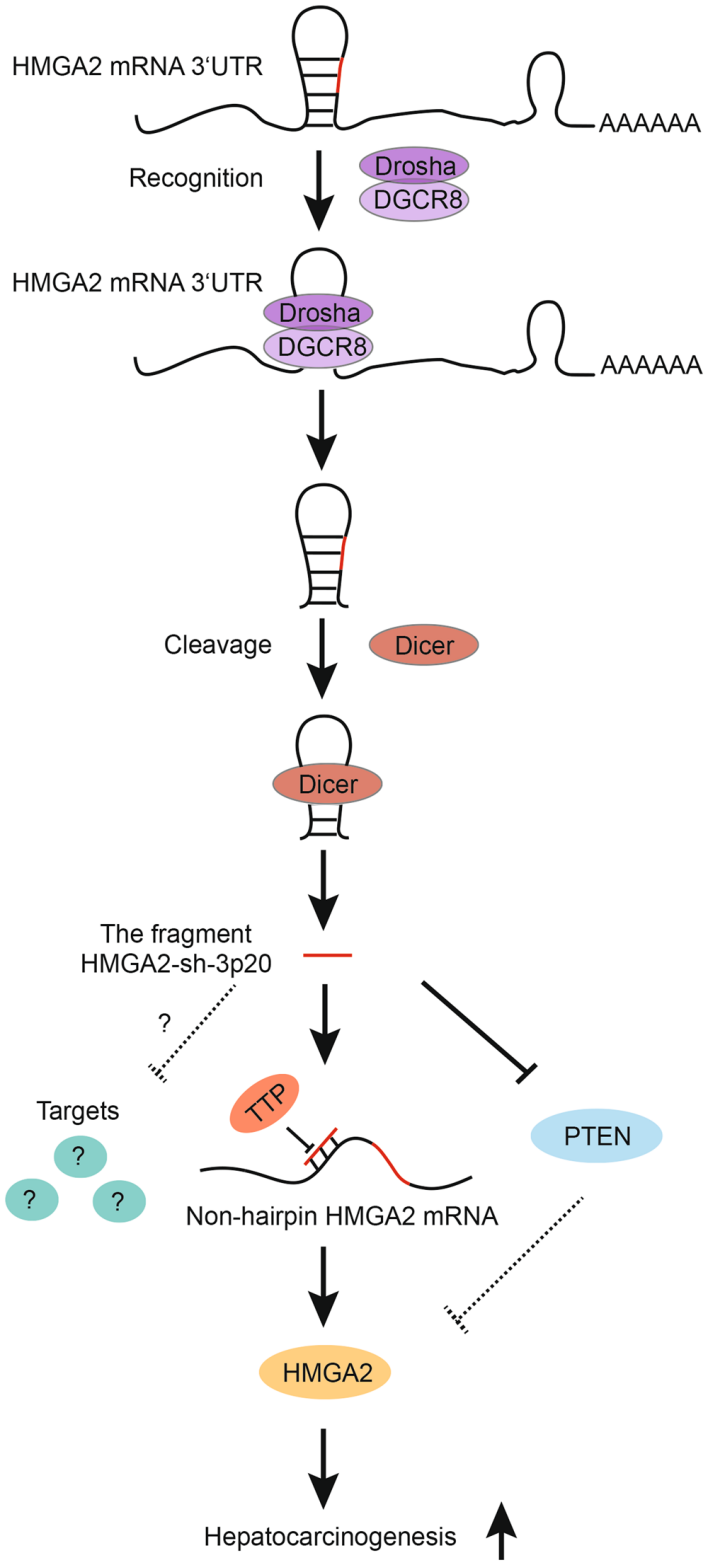
A model shows that the fragment HMGA2-sh-3p20 from HMGA2 mRNA 3′UTR promotes the growth of hepatoma cells by upregulating HMGA2. Bioinformatics analysis shows that 3′UTR of HMGA2 mRNA contains the hairpin structure (HMGA2-sh). Drosha and DGCR8 cleave the HMGA2-sh from the 3′UTR of HMGA2 mRNA, and Dicer contributes to the generation of the HMGA2-sh-3p20 from the HMGA2-sh. Furthermore, HMGA2-sh-3p20 is able to increase the levels of HMGA2 by antagonizing TTP-mediated HMGA2 degradation, while it decreases PTEN by targeting 3′UTR of PTEN mRNA. In addition, the downregulated-PTEN is not able to depress the expression of HMGA2, leading to the upregulation of HMGA2. Functionally, HMGA2-sh-3p20-enhanced HMGA2 accelerates the growth of liver cancer cells.

In summary, we present a model that HMGA2 enhances hepatocarcinogenesis (Fig. 7). Bioinformatics analysis shows that 3′UTR of HMGA2 mRNA contains the hairpin structure, termed HMGA2-sh. Drosha and DGCR8 cleave the HMGA2-sh from the 3′UTR of HMGA2 mRNA, and Dicer contributes to the generation of the HMGA2-sh-3p20 from the HMGA2-sh. Furthermore, HMGA2-sh-3p20 modulates its targets at the post-transcriptional level. Interestingly, HMGA2-sh-3p20 is able to increase the levels of HMGA2 by antagonizing TTP-mediated HMGA2 degradation, while it decreases PTEN by targeting 3′UTR of PTEN mRNA. In addition, the downregulated-PTEN is not able to depress the expression of HMGA2, leading to the upregulation of HMGA2. Functionally, HMGA2-sh-3p20-enhanced HMGA2 accelerates the growth of liver cancer cells. Thus, we conclude that fragment HMGA2-sh-3p20 from HMGA2 mRNA 3′UTR promotes the growth of hepatoma cells by upregulating HMGA2.

## Materials and Methods

### Cell Culture and Transfection

The human hepatoma cell lines HepG2, Huh7 and human kidney epithelial (HEK) 293T cells were purchased from the Cell Bank of Type Culture Collection of the Chinese Academy of Sciences, Shanghai Institute of Cell Biology. HepG2, Huh7 and 293T cells were maintained in DMEM (Gibco, USA), supplemented with heat-inactivated 10% FBS (Gibco, USA), 100 U/mL penicillin, and 100 μg/mL streptomycin and grown at 37 °C with 5% CO_2_ in a humidified incubator. The siRNAs targeting Dicer and TTP were reported previously^14, 40^. Si-Dicer, si-TTP and their negative control siRNA (si-NC), miR-150, miR-132, HMGA2-sh-3p20, HMGA2-sh-3p20 inhibitor and their respective negative controls were synthesized from Ribobio (Guangzhou, China). All sequences are listed in Supplementary Table S2. Cell transfection was performed using Lipofectamine 2000 (Invitrogen, USA) according to manufacturer’s protocol. After 48 h transfection, cells were collected for further experiments.

### Patient Samples

Thirty-five HCC tissue samples and their corresponding peritumor liver tissues were obtained from Tianjin First Center Hospital and Tianjin Tumor Hospital (Tianjin, China) after surgical resection. Written consent approving the use of tissue samples for research purposes was obtained from patients. Informed consent for study participation was also obtained from each patient. The information of patients with HCC is presented in Supplementary Table S3. The study protocol was approved by the Institute Research Ethics Committee at Nankai University. All experiments were performed strictly in accordance with relevant guidelines and regulations.

### Plasmid Constructions

A hairpin-contained fragment of HMGA2 3′UTR **(**position 3709-3934**)** was amplified by PCR from the cDNA of HepG2 using specific primers, which was further cloned into pRNAT-U6.1/neo vector to generate HMGA2-sh or cloned into pGL3-control vector to generate pGL-HMGA2. HMGA2-sh-mut was synthesized by Augct (Beijing, China) and was cloned into pRNAT-U6.1/neo vector to generate HMGA2-sh-mut. Two fragments of PTEN 3′UTR (nucleotides +3807 to +4141, +5137 to +5458) were cloned into pGL3-control vector *via* FseI/XbaI site to generate pGL-PTEN-1702 and pGL-PTEN-3062. Mutant construct of PTEN 3′UTR (named as pGL3-PTEN-1702-mut), carrying a substitution of 9 nucleotides within the paired sequence of HMGA2-sh-3p20, was conducted using overlapping extension PCR. The CDS region of TTP and PTEN were amplified by PCR from the cDNA of 293T cells using specific primers, and then was cloned into the pCMV-Tag2B vector. All plasmid constructions were verified by sequencing. All primers are listed in Supplementary Table S4.

### Luciferase Reporter Gene Assays

Luciferase reporter gene assays were performed using the Dual-Luciferase Reporter Assay System (Promega, USA) according to the manufacturer’s instructions. Cells were plated into 24-well plates at 3 × 10^4^ cells per well and transiently co-transfected with HMGA2-sh, HMGA2-sh-3p20 or their negative control and pGL-HMGA2 or pGL-PTEN or pGL3-control. The luciferase constructs of AP-1 (or NF-κB) contains transcriptional factor AP-1 (or NF-κB) binding element in the promoter region of pGL3-Basic^30, 41^. The luciferase activity of AP-1 (or NF-κB) can be influenced by the change of Ap-1 (or NF-κB) when the plasmid was transfected into the cells. The pRL-TK plasmid (Promega, USA) containing the Renilla luciferase gene was used for internal normalization.

### RNA extraction, RT-PCR and Quantitative RT-PCR

Total RNA was extracted from the cells (or tissues) using Trizol reagent (Invitrogen, USA) according to the manufacturer’s protocol. 1 μg of RNA was reverse transcribed into cDNA by ImPro-IIReverse Transcriptase (Promega, USA) following the manufacturer’s instruction. Following PCR amplification (30 cycles), PCR products were loaded on a 1.5% agarose gel. To examine the expression of mRNAs, qRT-PCR was performed according to the instructions of Fast Start Universal SYBR Green Master (Rox) (Roche, Germany). GAPDH was applied as an internal control to normalize HMGA2 and PTEN mRNA levels. U6 was applied as an internal control to normalize the expression level of HMGA2-sh-3p20. All primers are listed in Supplementary Table S4. Threshold cycle (Ct) changes was calculated by ΔCt = Ct (target) − CT (control) and fold changes for target gene was calculated as 2^−Δ (ΔCt)^. Data was graphed and analyzed using the GraphPad Prism software package.

### HMGA2-sh-3p20 Detection and PCR Analysis

Total RNA was extracted from 293T cells using Trizol reagent (Invitrogen, USA) according to the manufacturer’s protocol. Then, 2 μg RNA was polyadenylated by poly (A) polymerase (Ambion, USA) as described previously^42^. According to the manufacturer’s instruction, 1 μg polyadenylated RNAs were reverse transcribed into cDNA by ImPro-IIReverse Transcriptase (Promega, USA) using reverse transcription primer (5′-GCGAGCACAGAATTAATACGACTACTATAGGTTTTTT TTTTTTTTTTTTVN-3′). The primers for PCR are listed in Supplementary Table S4. The procedure of PCR is denaturation at 95 °C for 5 min; 35 cycles at 95 °C for 20 s, 57 °C for 20 s and 72 °C for 6 s; 72 °C for 5 min. U6 was used as an internal control to normalize the expression level of HMGA2-sh-3p20. The PCR products were ran on a 3% agarose gel. Then, the PCR product of HMGA2-sh-3p20 was cloned into pEASY-T1 vector (TransGen, China) and the constructs were verified by sequence analysis.

### Western Blot Analysis

Western blot analysis was carried out with standard protocols^43^. Protein was extracted from cultured cells or tissue samples using RIPA lysis (Solarbio, China) following the manufacturer’s instructions. The protein samples were run on 12% polyacrylamide gel and transferred to PVDF membranes. Then, membranes were blocked in 8% nonfat dry milk at room temperature for 2 h and then incubated with primary antibody at 4 °C for overnight. The dilution of primary antibody is following: HMGA2 (1: 5000, Genetex), PTEN (1:800, Proteintech), TTP (1:1000, Proteintech), Drosha (1:1000, Proteintech), DGCR8 (1:800, Proteintech), Dicer (1:800, Proteintech), β-actin (1:2000, Abcam). Membranes were washed in PBS-Tween and incubated with secondary antibodies anti-rabbit (1:5000, Santa Cruz) or anti-mouse (1:5000, Santa Cruz). Subsequently, blots were detected by using ECL assay kit (GE Healthcare Life Science). All antibodies are listed in Supplementary Table S5.

### RNA Immunoprecipitation Assays

RIP assays were performed in native conditions as described^44^. Briefly, HepG2 cells were pelleted and lysed. Then, the lysates were passed through a 27.5 gauge needle 4 times to facilitate nuclear lysis. The supernatant was incubated with Drosha or DGCR8 antibody or IgG at 4 °C for overnight. Next, RNA/antibody complex was incubated with protein-A beads (Sigma, USA) at 4 °C for 2 hours. The RNA/antibody/protein-A beads complex was washed six times by NT2 buffer (50 mM Tris-HCl pH 7.4, 150 mM NaCl, 1 mM MgCl_2_, 0.05% NP-40). The RNA was extracted with Trizol (Invitrigen, CA) based on the manufacturer’s protocol and subjected to RT-PCR analysis.

### Immunohistochemistry Staining

The tumor tissues from nude mice were fixed and embedded with paraffin after those mices were sacrificed. Immunohistochemistry staining was performed as previously reported^45^. In brief, the slides were deparaffinized and rehydrated. Then, antigen retrieval was applied at 95 °C with citrate buff (pH 6.0) for 15 min. The slides treated with 3% H_2_O_2_ for 10 minutes and blocked with goat serum for 1 h. Next, the slides incubated with rabbit anti-Ki67 (1:200, Thermo) at 4 °C for overnight. Subsequently, the slides were incubated with horseradish peroxidase labelled anti-rabbit at room temperature for 30 min. Immunostaining was proceeded using chromogen 3, 3′-Diaminobenzidine (DAB) and counter stained with Mayer’s hematoxylin (ZSBG-BIO, China). The slides were then dehydrated and covered with coverslip.

### Determination of HMGA2 mRNA stability

The mRNA stability determinations were performed as previous reports^46^. In brief, HepG2 cells were transfected with HMGA2-sh-3p20 or mimics control. After 12 h, these cells were incubated with 10 µg/mL actinomycin D (Act-D, Sigma) for 4, 8 or 12 h. Then, total RNAs were extracted and the HMGA2 mRNA were examined by qRT-PCR analysis and normalized by GAPDH. The mRNA half-life was predicted from the HMGA2 mRNA decay curve.

### Cell Proliferation Assays

For quantitative proliferation assays, HepG2 or Huh7 cells were seeded onto 96-well plates (1000 cells/well) for 12 h before transfection and methyl thiazolyl tetrazolium (MTT) (Sigma, USA) assays were carried out as described previously^47^. Briefly, cells were cultured after different days and 15 μL of MTT was added to each well, followed by incubation for 4 hours. The supernatant was discarded and 100 μL of dimethyl sulfoxide was added to stop the reaction. Absorbance at 490 nm was assessed using an ELISA reader system (Labsystem, Multiskan Ascent). The Ethynyldeoxyuridine (EdU) incorporation assay was performed using the Cell-Light TM EdU imaging detecting kit according to the manufacturer’s instructions (RiboBio, Guangzhou, China).

Flow cytometry analysis was processed as earlier described^48^. HepG2 or Huh7 cells were transfected with HMGA2-sh-3p20. After 48 h transfection, the cells were collected and fixed in 70% ethanol at 4 °C for overnight. Then, the fixed cells were resuspended in propidium iodine (PI) solution, including 50 mg/mL PI (Biorbyt, China) and 50 mg/mL RNaseA (Sigma, USA) in PBS, and incubated at 37 °C for 30 minutes. Stained cells were filtered with a nylon-mesh sieve. Next, the cells were analyzed by a FACScan flow cytometer and analysised by Cell Quest software (Becton Dickinson, USA). For colony formation assays, HepG2 or Huh7 cells were transfected with 100 nM HMGA2-sh-3p20 or mimics NC, respectively. 48 h after transfection, 1000 cells were plated into 6-well plates and kept in complete medium for 2 weeks. Colonies were fixed with methanol and stained with methylene blue or crystal violet^49^.

### *In Vivo* Tumorigenicity Assays

All experimental procedures involving animals were in accordance with the Guide for the Care and Use of Laboratory Animals (NIH publications nos. 80–23, revised 1996) and were performed according to the institutional ethical guidelines for animal experiment. All experimental were approved by the Institute Research Ethics Committee at Nankai University. In brief, HepG2 cells were transfected with 100 nM HMGA2-sh-3p20 or mimics NC using Lipofectamine RNAiMAX (Invitrogen, Carlsbad, CA). At 48 hours after transfection, HepG2 cells were resuspended with sterile PBS and 10^7^ viable cells were subcutaneously injected into 4-week-old male BALB/c athymic nude mice (Experiment Animal Center of Peking, China; each group, n = 6). Tumor growth was measured beginning 5 days after injection of hepatoma cells. Tumor volume (V) was monitored by measuring the length (L) and width (W) of the tumors with calipers and was calculated using the formula (L × W^2^) × 0.5. After 30 days, all mices were sacrificed. Tumor weight and the expression of HMGA2 and PTEN were measured.

### Statistical Analysis

Each experiment was repeated at least three times. Statistical significance was assessed by comparing mean values ( ± SD) using the Student’s *t* test for independent groups as follow: *P < 0.05; **P < 0.01; ***P < 0.001 and not significant (NS). The Pearson correlation coefficient was used to determine the correlations among gene expression in tumor tissues. The expression of HMGA2-sh-3p20 in tumor tissues and matched peritumor tissues were compared using the Wilcoxon signed-rank test.

## Electronic supplementary material

Supplemental Information
