# Supplementary material for: The Fragment HMGA2-sh-3p20 from HMGA2 mRNA 3′UTR Promotes the Growth of Hepatoma Cells by Upregulating HMGA2
Source: Sci Rep. 2017 May 18;7:2070. doi: 10.1038/s41598-017-02311-0 (PMC5437003; doi:10.1038/s41598-017-02311-0)
Supplement: Supplementary file 1 — Supplemental Information [file 41598_2017_2311_MOESM1_ESM.pdf]

The Fragment HMGA2-sh-3p20 from HMGA2 mRNA 3′UTR Promotes the Growth of Hepatoma Cells by Upregulating HMGA2

Yuan Wang[1], Fuquan Chen[1], Zhe Yang[1], Man Zhao[1], Shuqin Zhang[1], Yuen Gao[1], Jinyan Feng[1], Guang Yang[1], Weiying Zhang[1], Lihong Ye[2*] and Xiaodong Zhang[1*]

[1] State Key Laboratory of Medicinal Chemical Biology, Department of Cancer Research, College of Life Sciences, Nankai University, Tianjin 300071, China.

[2] State Key Laboratory of Medicinal Chemical Biology, Department of Biochemistry, College of Life Sciences, Nankai University, Tianjin 300071, China.

[*] Correspondence and requests for materials should be addressed to X.Z. (email: zhangxd@nankai.edu.cn.) or L.Y. (e-mail: yelihong@nankai.edu.cn.)

Supplementary Information:

Supplementary Figures

Supplementary Figures Legends

Supplementary Tables

Supplementary Figure S1. A hairpin within HMGA2 mRNA 3'UTR has regulatory function

A

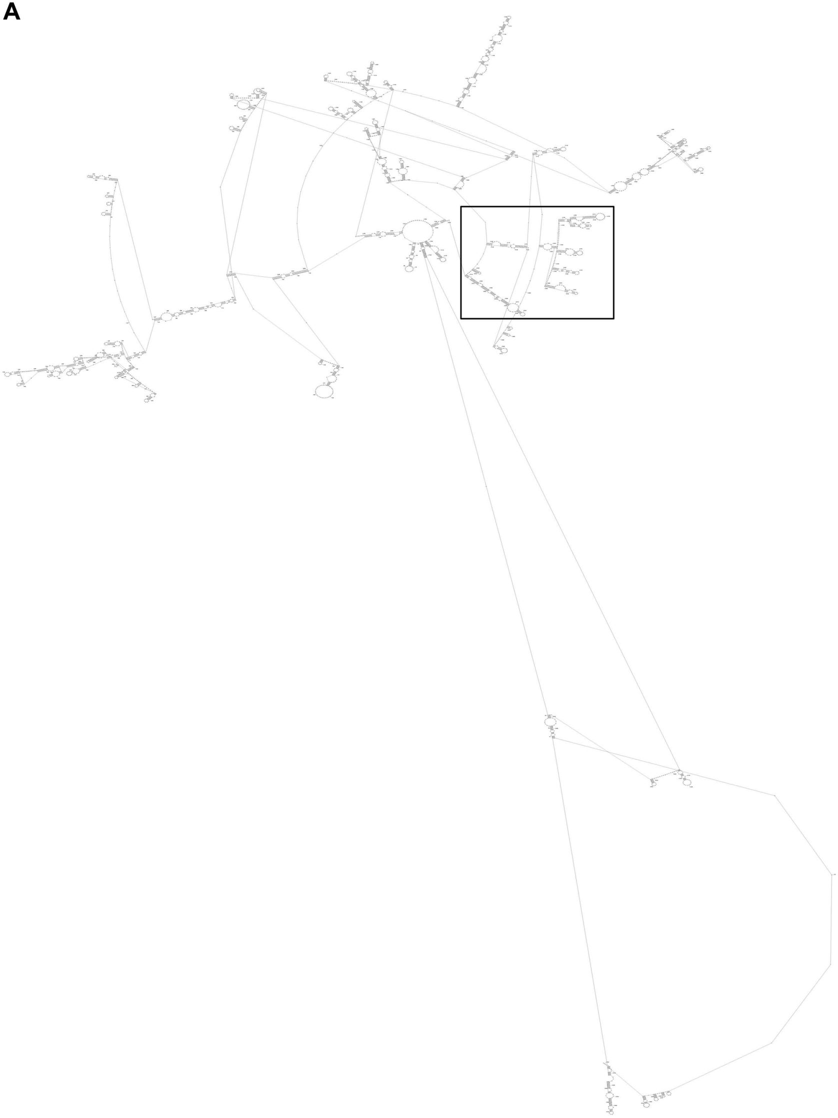

Supplementary Figure S1. A hairpin within HMGA2 mRNA 3'UTR has regulatory function

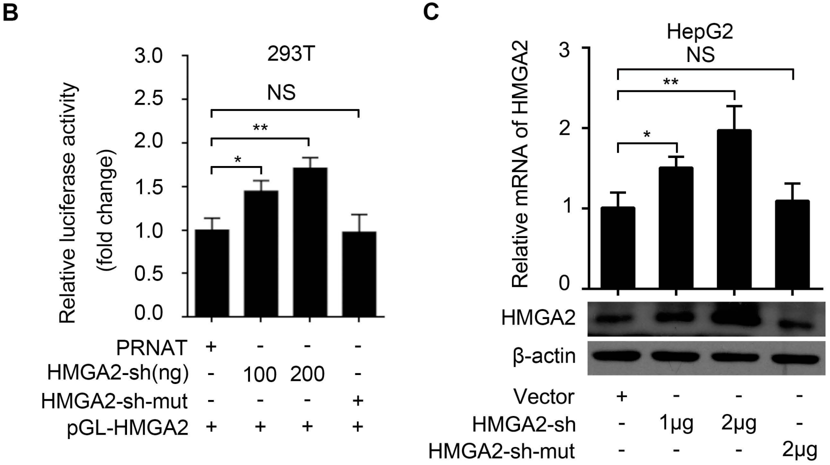

Supplementary Figure S2. HMGA2-sh-3p20 is cleaved from HMGA2-sh by Drosha/DGCR8 complex and Dicer

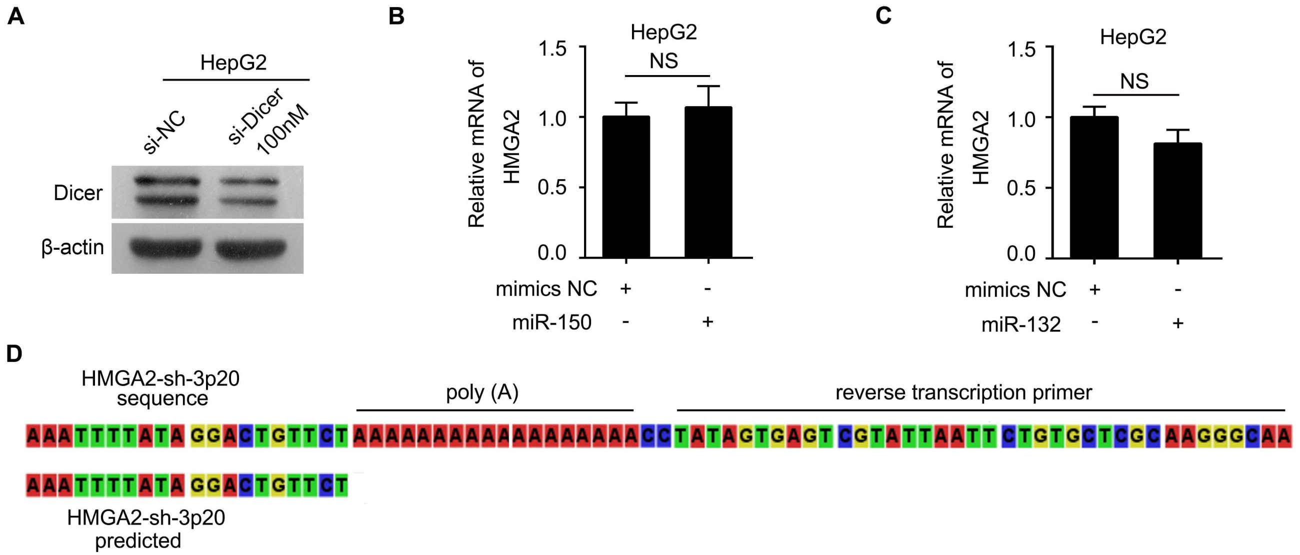

Supplementary Figure S3. HMGA2-sh-3p20 upregulates HMGA2 by blocking the TTP-mediated degradation of HMGA2 mRNA

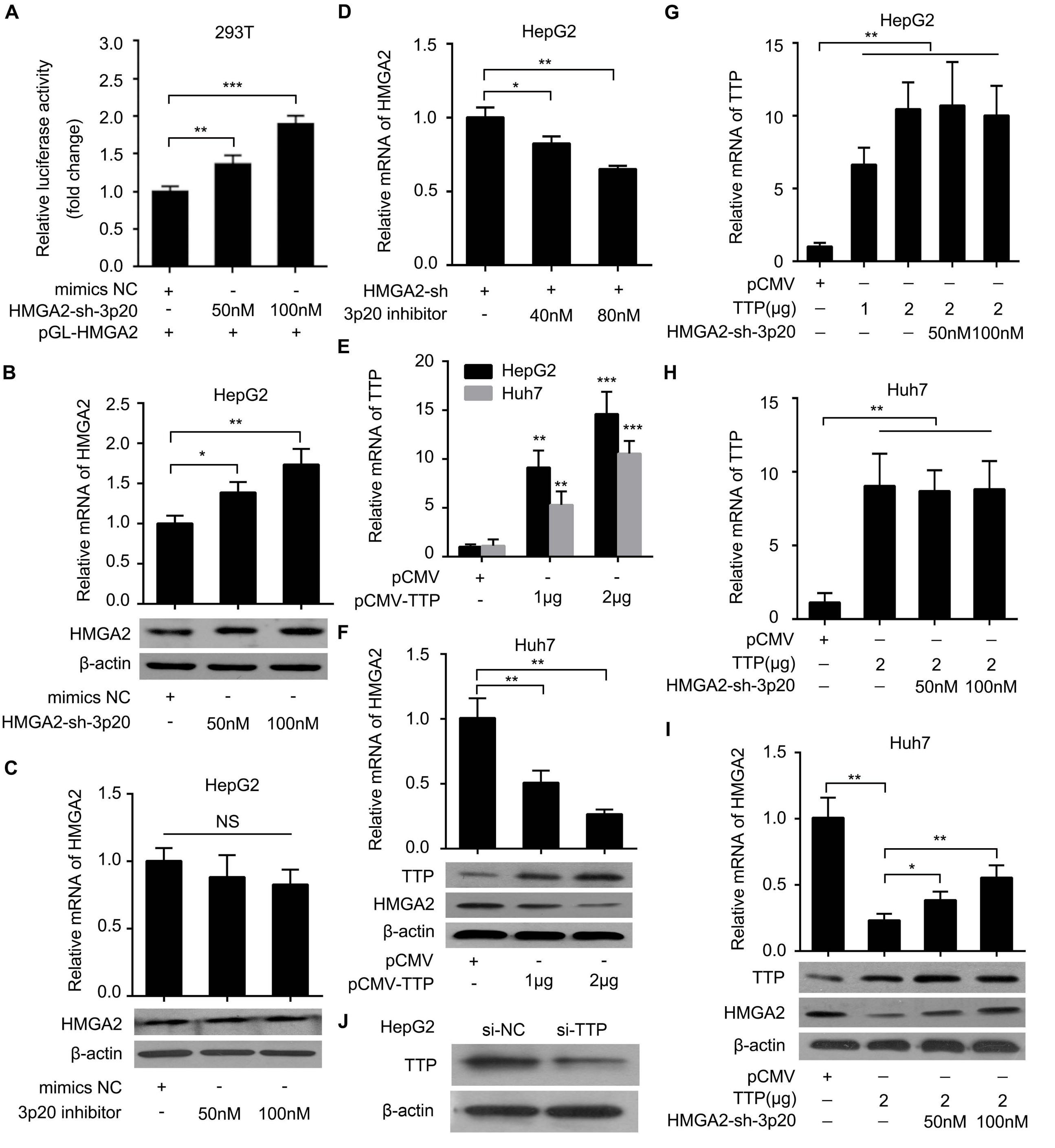

Supplementary Figure S4. HMGA2-sh-3p20 inhibits PTEN by targeting the 3'UTR of PTEN mRNA

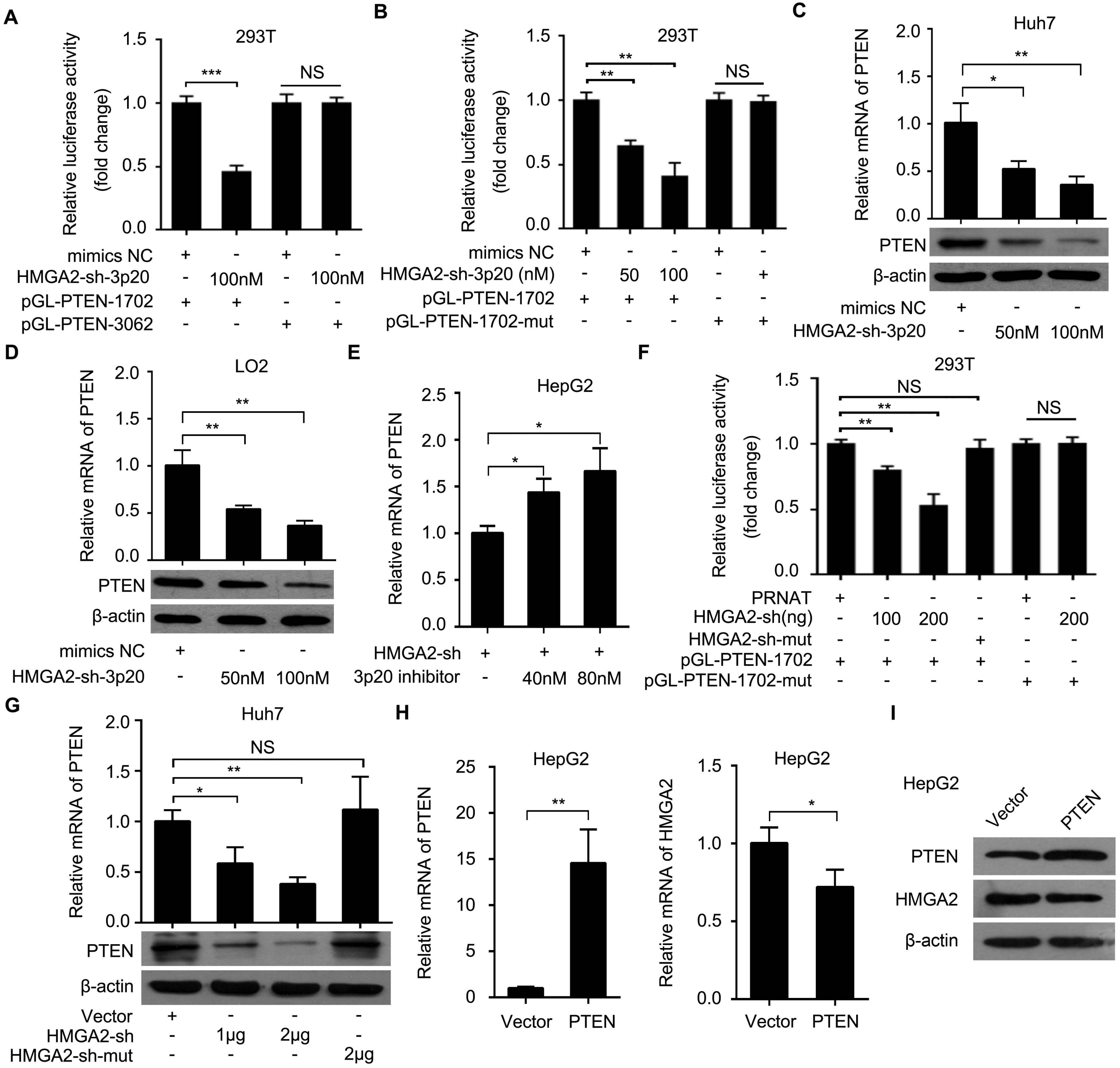

Supplementary Figure S5. HMGA2-sh-3p20 contributes to the proliferation of hepatoma cells

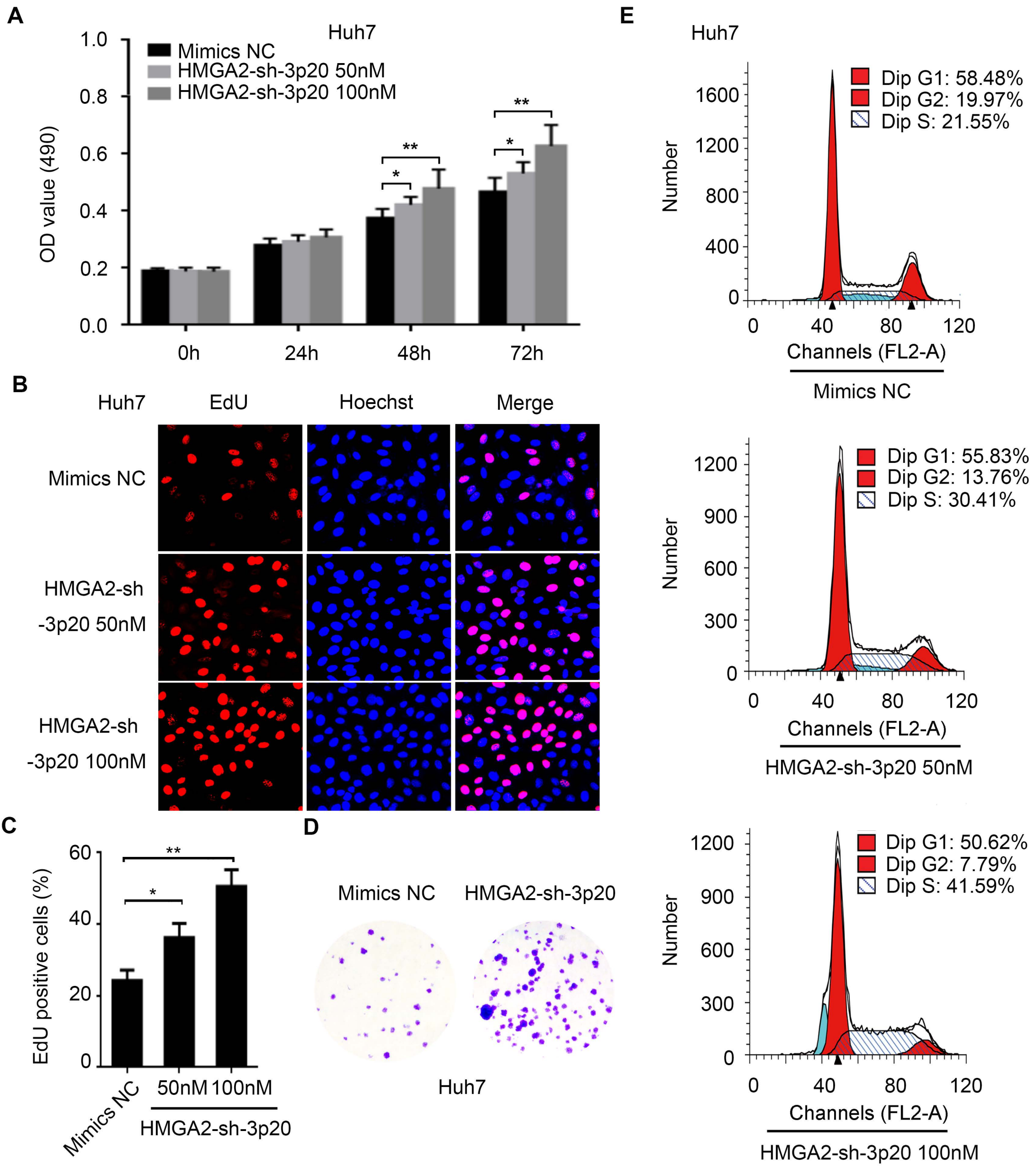

Supplementary Figure 6. Full bands for walking PCR in Figure 2

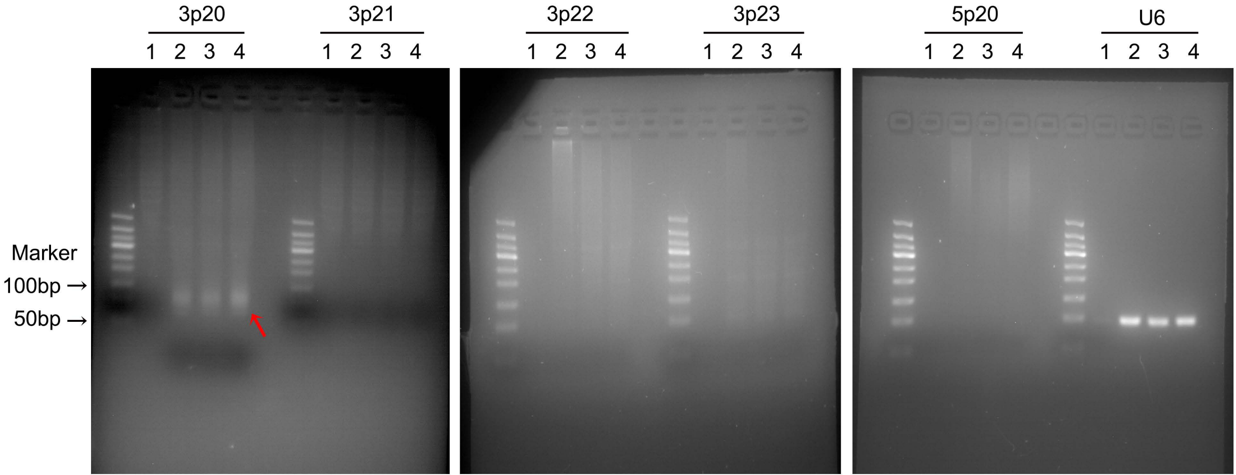

Supplementary Figure 7. Full Blots in Figure 3

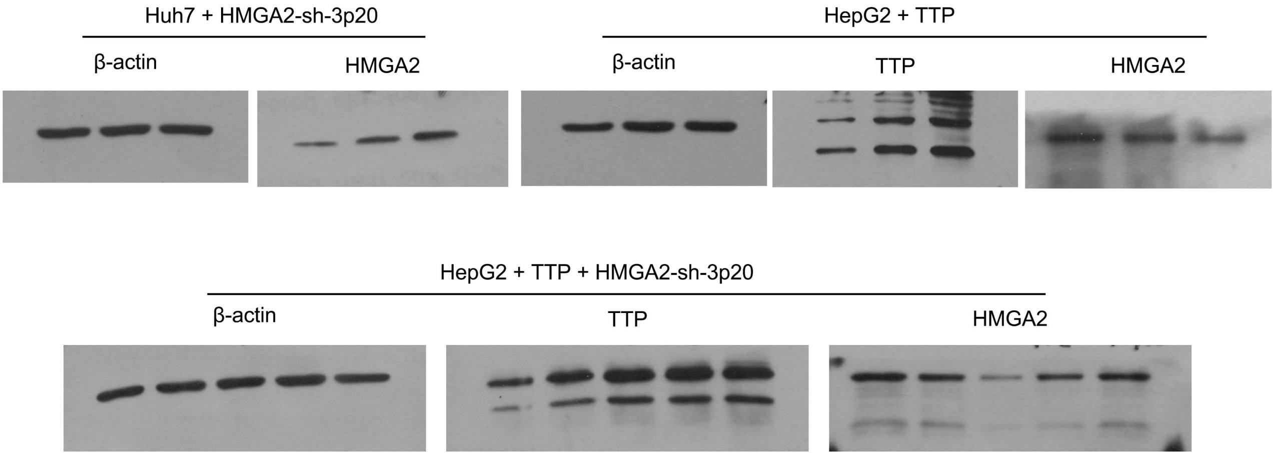

Supplementary Figure Legends

Supplementary Figure S1. **A hairpin within HMGA2 mRNA 3′UTR has regulatory function.** (A) The secondary structure of HMGA2 3′UTR was analyzed using RNAstructure. (B) The luciferase activities of pGL3-HMGA2 were examined by luciferase reporter gene assays in 293T cells. (C) The expression of HMGA2 was assessed by qRT-PCR and Western blot analysis in HepG2 cells. Every experiment was repeated three times. Error bars represent s.d. (n=3), $^{**}p<0.01$;$^{***}p<0.001$, Student's *t* test.

Supplementary Figure S2. **HMGA2-sh-3p20 is cleaved from HMGA2-sh by Drosha/DGCR8 complex and Dicer.** (A) The interference efficiency of si-Dicer was validated by Western blot analysis in HepG2 cells. (B and C) Effect of miR-150 (B) and miR-132 (C) on the levels of HMGA2 mRNA was assessed by qRT-PCR in HepG2 cells. (D) The sequence analysis of HMGA2-sh-3p20 PCR product.

Supplementary Figure S3. **HMGA2-sh-3p20 upregulates HMGA2 by blocking the TTP-mediated degradation of HMGA2 mRNA.** (A) The luciferase activities of pGL3-HMGA2 were measured by luciferase reporter gene assays in 293T cells. (B) Effect of HMGA2-sh-3p20 on the expression of HMGA2 was detected by qRT-PCR and Western blot in HepG2 cells. (C) Effect of HMGA2-sh-3p20 inhibitor on the expression of HMGA2 was assessed by qRT-PCR and Western blot in HepG2 cells. (D) The expression of HMGA2 was examined by qRT-PCR in HepG2 cells transiently co-transfected with HMGA2-sh and

HMGA2-3p20 inhibitor. (E) The expression of TTP was validated by qRT-PCR in HepG2 and Huh7 cells transiently transfected with TTP, respectively. (F) The expression of HMGA2 was measured by qRT-PCR and Western blot in Huh7 cells. (G and H) The expression of TTP was validated by qRT-PCR in HepG2 and Huh7 cells, respectively. (I) Effect of HMGA2-sh-3p20 on the expression of TTP-mediated HMGA2 was assessed by qRT-PCR and Western blot in Huh7 cells. (J) The interference efficiency of si-TTP was validated by Western blot analysis in HepG2 cells. Every experiment was repeated three times. Error bars represent s.d. (n=3), **p<0.01;***p<0.001, Student's *t* test.

Supplementary Figure S4. **HMGA2-sh-3p20 inhibits PTEN by targeting the 3′UTR of PTEN mRNA.** (A) The luciferase activities of pGL3-PTEN-1702 and pGL3-PTEN-3062 were measured by luciferase reporter gene assays in 293T cells. (B) Effect of HMGA2-sh-3p20 on the luciferase activities of pGL3-PTEN-1702 and pGL3-PTEN-1702-mut were assessed by luciferase reporter gene assays in 293T cells. (C and D) Effect of HMGA2-sh-3p20 on the expression of PTEN was examined by qRT-PCR and Western blot in Huh7 and LO2 cells, respectively. (E) The expression of PTEN was assessed by qRT-PCR in HepG2 cells transiently co-transfected with HMGA2-sh and HMGA2-3p20 inhibitor. (F) Effect of HMGA2-sh on the luciferase activities of pGL3-PTEN-1702 and pGL3-PTEN-1702-mut were measured by luciferase reporter gene assays in 293T cells. (G) Effect of HMGA2-sh on the expression of PTEN was assessed by qRT-PCR and Western blot in Huh7 cells. (H) The transfection efficiency of PTEN was validated by qRT-PCR in HepG2 cells. Effect of PTEN on the levels of HMGA2 mRNA was examined by qRT-PCR in HepG2

cells. (I) Effect of PTEN on the expression of HMGA2 was measured by Western blot in HepG2 cells. Every experiment was repeated three times. Error bars represent s.d. (n=3), ***p*<0.01;****p*<0.001, Student's *t* test.

Supplementary Figure S5. **HMGA2-sh-3p20 contributes to the proliferation of hepatoma cells.** (A) Effect of HMGA2-sh-3p20 on proliferation of Huh7 cells was determined by MTT assays. (B and C) Effect of HMGA2-sh-3p20 on proliferation of Huh7 cells was measured by EdU incorporation assays. (D) Effect of HMGA2-sh-3p20 on proliferation of Huh7 cells was measured by colony-formation assays. (E) Effect of HMGA2-sh-3p20 on proliferation of Huh7 cells was tested by flow cytometry assays. Every experiment was repeated three times. Error bars represent s.d. (n=3), ***p*<0.01;****p*<0.001, Student's *t* test.

Supplementary Figure S6. Full bands images. Full bands images for PCR in Figure 2G. The template is purified water or cDNA of 293T cells transfected with different plasmid. 1, purified water; 2, without plasmid; 3, empty vector plasmid; 4, PRNAT-HMGA2-sh plasmid.

Supplementary Figure S7. Full blots images. Full blots images for Figure 3D, H and I.

Supplementary Table

Supplementary Table S1. 3′UTR of mRNAs derived hairpins prediction.

No	gene	genebank ID	sequence
1	HMGA2	NM_003483.4	AGCTTGTGGCCAATGGAACAGTAAGAACATCATAAAATTTTTATATATATAGTTTATTTTTGTGGGAGATAAATTTTATAGGACTGTTCTTTGCTGTTGTTGGTCGCAGCTACATAAGACTGGACATTT
2	TLR8	NM 138636.5	CAGTCCAGGGAAAACAGCTGAAGACCAAGATGGTGAGCTCTGATTGCTTCAGTTGGTCATCAACTATTTTCCCTTGACTG
3	RAC1	NM_006908.4	GTGTTGCAGCTTTATAGTTTTTAAAATATTTTAGATAATTCTTAAACTATGAACCTTCTTAACAT
4	PI3K	NM_006218.2	TTAATGAAGAAAAATGCTTGGGGTGGAAGGGACTCTTGAGATTTCACCAGAGACTTTTTCTTTTTAATAAATCAAACCTTTTGATGATTTGAGGTTTTATCTGCAGTTTT
5	beclin	NM_003766.3	GGTTGGGCCGCCTCCCCACTGTCCGTGTGGGCTGGAATGAGATTAACACTGCCTGGGGACAGGCGGCCT
6	SRC	NM_005417.4	GTGATGTTTGACCTTCAGAGCCAGCCGGCTATGAAAGGGAGCGAGCCCCTCGGCTCTGGAGGCAATCAAGCAGACAT
7	JUN	NM_002228.3	TTGGGGAGGGGCGGCAGGAGGGAGGTTTGTGAGAGCGAGGCTGAGCCTACAGATGAACTCTTTCTGGCCTGCCTTCGTTAA
8	LATS	NM_001270519.1	GCTGTGCTGCTCCGCAGACCACCGCCTGGGGCGGAATGGGGCCGATGACCTGAAGGCCCACCCCTTCTTCAGCGCCATTGACTTCTCCAGTGACATCGGAAGCAGCCAGC
9	CTBP1	NM_001012614.1	CAACAAAAAGAAAACCTGAAGGAGCATTTGGAAGTCAATTTGAGGTTTTTTTTTTTGTT
10	CEBPB	NM_001285878.1	CGCGGCCCCCGCGCGCGTCCCCCTGCCGGCCGGGGCTGAGACTCCGGGGAGCGCCCGCGCCCGCGCCCTCGCCCCCGCCCCCGGCGGCGCCGGCAAAACTTTGGCACTGGGGCACTTGGCAGCGCGGGGAGCCCG
11	VASP	NM_003370.3	AGCTATTTAAGGGGAGGGGATGTCTCACCGGGCTGGGGGTGAGATATCCCCCCACCCCAGGGACTCCCCTTCCCTCTGGCT
12	DUSP5	NM_004419.3	CCTCCGAGGCCTGCGCGACCCACCTACACTACAAATGGATCCCTGTGGAAGACAGCCACACGGCTGACATTAGCTCCCACTTTCAAGAAGCAATAGACTTCATTGACTGTGTCAGGGAAA
13	PABP	NM 002568.3	ATTTTGTAATTCTTTACTGTGGAATAGCTCAGAATGTCAGTTCTGTTTTAAGTAACAGAATTGATAACTGAGCAAGGAAACGTAATTTGGATTATAAAAT

14	CTNNBIP1	NM_001012329.1	GTGTGCATGTGCTGTGTGAGTGAGCACACCCGTGTGCACACTCATACACATGTGCACACGTGTGCATGTGCTGTGTGAGTGAGCACACCCGTGTGCACACTCATACACATGTGCACACA
15	CASP3	NM_004346.3	ATGATGACATGGCGTGTCATAAAATACCAGTGGAGGCCGACTTCTTGTATGCATACTCCACAGCACCTGGTTATTA
16	E2F1	NM_005225.2	GTCCCACAAGGCCCAGGGCAGTGCCTGCTCCCAGAATCTGGTGCTCTGACCAGGCCAGGTGGGGAGGCTTTGGCTGGCTGGGCGTGTAGGAC
17	MSL2	NM_001145417.1	AGTCTTTATTTTGAATATGCTCCTAGTTTTTTTTTAGGGTGCTGTTCATTATGAAGGCTTCTTTATAGAGGCCTAATAAGAATGCCTTTTTATAAAGCCTGTGCATTTAGGTAGGTTGA
18	IL-6	NM_000600.3	ATTTCTTGGAAAGTGTAGGCTTACCTCAAATAAATGGCTAACTTATACATATTTTTAAAGAAATATTTATATTGTATTTATATAATGTATAAATGGTTTTTATACCAATAAATGGCATTTTAAA
19	NIK	NM_003954.4	CCCTGCCCTGCCCCTGGCCAGAGGGTACTGCCGACGGCACTTTGCACTCTGATGACCTCAAAGCACTTTCATGGCTGCCCTCTGGCAGGGCAGGG
20	c-Fos	NM_005252.3	TTTTTATTTTATTTTTTTCUACCTTGAGGTCTTTTGACATGTGGAAAGTGAATTTGAATGAAAA
21	IP3R	NM_001099952.2	AGTTTTATACTGTATGTATATGATTGCTACTCTAAAGGTTTGGATATATGTATTGTAATTAGAATTG
22	GRB2	NM_002086.4	None
23	LIN28A	NM_024674.4	None
24	HIF1A	NM_001243084.1	None
25	BCL2	NM_000633.2	None
26	NFKB2	NM_001077494.3	None
27	KRAS	NM_004985.4	None
28	MTOR	NM_004958.3	None
29	PRRT2	XM_006721006.1	None
30	PIK3CA	NM_006218.2	None
31	RBFOX1	NM_001142333.1	None
32	RBFOX2	NM_001031695.2	None
33	BID	NM_001196.3	None
34	MAP3K14	NM_003954.4	None
35	IKBKB	NM_001190720.2	None
36	EEF2K	NM_013302.3	None
37	RICTOR	NM_001285439.1	None
38	PPARA	NM_001001928.2	None
39	TESK2	NM_007170.2	None
40	CAT	NM_001752.3	None

41	TRIP10	NM_001288962.1	None
42	WAS	NM_000377.2	None
43	TIAM1	NM_003253.2	None
44	PTK2	NM_001199649.1	None
45	NCK2	NM_001004720.2	None
46	NCK1	NM_001190796.1	None
47	FASCIN1	NM_003088.3	None
48	MYLK	NM_053025.3	None
49	MLC1	NM_015166.3	None
50	CAP2	NM_006366.2	None
51	TMOD1	NM_001166116.1	None
52	DSTN	NM_001011546.1	None
53	PDXP	NM_020315.4	None
54	SSH3	NM_017857.3	None
55	KRIT1	NM_001013406.1	None
56	FLNC	NM_001127487.1	None
57	FLNB	NM_001164317.1	None
58	TRIO	NM_007118.2	None
59	CIR1	NM_004882.3	None
60	RHOA	NM_001664.2	None
61	AKT1	NM_001014431.1	None
62	F2RL2	NM_001256566.1	None
63	ZBTB4	NM_001128833.1	None
64	CSNK2A1	NM_001895.3	None
65	NKD2	NM_001271082.1	None
66	TAB1	NM_006116.2	None
67	TAB2	NM_015093.4	None
68	RARA	NM_000964.3	None
69	CSNK1A1	NM_001025105.2	None
70	SFRP1	NM_003012.4	None
71	PPOX	NM_000309.3	None
72	FYN	NM_002037.5	None
73	CSNK2B	NM_001282385.1	None
74	CAMK	NM_001204492.1	None
75	EGR1	NM_001964.2	None
76	MEF2C	NM_002397.4	None
77	JNK1	NM_002750.3	None
78	JNK2	NM_139068.2	None
79	MKK3	NM_002756.4	None
80	MKK4	NM_003010.3	None
81	MKK6	NM_002758.3	None
82	RAP1	NM_001010935.1	None
83	VAV	NM_005428.3	None

84	Bam32	AF186022.1	None
85	CD19	NM_001178098.1	None
86	CD22	NM_001771.3	None
87	SHP-1	NM_002831.5	None
88	LYN	NM_002350.3	None
89	SYK	NM_003177	None
90	BTK	NM_000061.2	None
91	MALT1	NM_006785.3	None
92	CARMAL	NM_032415.5	None
93	Stim1	NM_001277961.1	None
94	Orai1	NM_032790.3	None
95	Calpain	AJ242832.1	None
96	Zap70	NM_001079.3	None
97	LCK	NM_001042771.2	None
98	REI	NM_001123041.2	None
99	CAMK4	NM_001744.4	None
100	p65	AJ002425.2	None
101	ABCC2	NM_000392.3	None
102	ATF4	NM_001675.4	None
103	CAST	NM_001042440.3	None
104	CCNA1	NM_001111045.1	None
105	CD99	NM_001122898.1	None
106	CYBB	NM_000397.3	None
107	DOK1	NM_001197260.1	None
108	EDN1	NM_001168319.1	None
109	FOS	NM_005252.3	None
110	GABRE	NM_004961.3	None
111	HSPA5	NM_005347.4	None
112	MAP2K2	NM_030662.3	None
113	MCM6	NM_005915.5	None
114	MYH10	NM_001256012.1	None
115	ORC3	XM_005248704.1	None
116	PIAS1	NM_016166.1	None
117	PIK3R4	NM_014602.2	None
118	PRKDC	NM_001081640.1	None
119	SMAD6	NM_001142861.2	None
120	WNT5B	NM_030775.2	None
121	FOXO4	NM_001170931.1	None
122	PDE3A	NM_000921.4	None
123	PDE2A	NM_002599.4	None
124	YARS	NM_003680.3	None
125	SOAT1	NM_003101.5	None
126	GSS	NM_000178	None

127	GOT2	NM_002080.3	None
128	HERC1	NM_003922.3	None
129	WWP1	NM_007013	None
130	NEDD4	NM_001284338.1	None
131	smad1	NM_005900	None
132	PP2A	L42373.1	None
133	Axin	NM_003502.3	None
134	APC	M74088	None
135	GSK-3	NM_019884.2	None
136	SARA	AF104304.1	None
137	NuMB	NM_001005743	None
138	KDM5A	NM_001042603.2	None
139	CSL	NM_005349.3	None
140	SKIP	AB036829.1	None
141	HAT	AK290289.1	None
142	O-Fut	BC000582.2	None
143	Furin	NM_002569.3	None
144	Notch	NM_017617	None
145	Ihh	NM_002181	None
146	shh	NM_000193	None
147	smad2	NM_001135937.2	None
148	smad3	NM_005902.3	None
149	smad4	NM_005359	None
150	smad5	NM_005903.5	None
151	smad6	BC052569.1	None
152	MAVS	NM 001206491.1	None
153	TLR9	NM 017442.3	None
154	TLR7	NM 016562.3	None
155	TLR3	NM 003265.2	None
156	TRIF	NM 182919.3	None
157	SOCS-1	NM 003745.1	None
158	IRAK-4	NM 001114182.2	None
159	IRAK-M	NM 001142523.1	None
160	MYD88	NM 001172566.1	None
161	TIRAP	NM 001039661.1	None
162	ELKS	NM 178039.2	None
163	TAX	NM 005076.3	None
164	COT	NM 001143935.1	None
165	CKII	NM 001895.3	None
166	smurf2	NM 022739.3	None
167	LIMK	NM 001204426.1	None
168	TF	NM 001063.3	None
169	BMI1	NM 005180.8	None

170	Rad51	NM 001164269.1	None
171	BORA	NM 001286746.1	None
172	AURA	NM 003600.2	None
173	P90RSK	NM 001006932.1	None
174	DNA-PK	NM 001081640.1	None
175	HRI	NM 001134335.1	None
176	GADD34	NM 014330.3	None
177	PDK3	NM 001142386.2	None
178	Raf	NM_014943.3	None
179	MDM2	NM_001145337.2	None
180	stat3	NM_003150.3	None
181	4-Oct	NM_001173531.2	None
182	MAPK	NM_002745.4	None
183	AP1	NM_002228.3	None
184	NOS	NM_000620.4	None
185	MP1	NM_001243736.1	None
186	ATF1	NM_005171.4	None
187	PCNA	NM_002592.2	None
188	NANOG	XM_005253484.2	None
189	KLF4	NM_004235.4	None
190	HBXIP	NM_006402.2	None
191	MYC	NM_002467.4	None
192	F3	NM_001178096.1	None
193	BCR	NM_004327.3	None
194	LEP	NM_000230.2	None
195	PTGS2	NM_000963.3	None
196	JAK2	NM_004972.3	None

Supplementary Table S2. List of siRNAs used in this paper.

**Gene**	**Sequence (5'- 3')**
si-Dicer	UUUGUUGCGAGGCUGAUUC
si-TTP	GACGGAACUCUGUCACAAG
si-NC	AAUGGUCAUGGUCUUAUUCC
HMGA2-sh-3p20	AAAUUUUAUAGGACUGUUCU
mimics NC	UUCUUCGAAGGUGUGACGU
HMGA2-sh-3p20 inhibitor	AGAACAGUCCUAUAAAAUUUA
HMGA2-sh-3p20 inhibitor NC	CAGUACUUUUGUGUAGUACAA
miR-150	UCUCCCAACCCUUGUACCAGUG
miR-132	UAACAGUCUACAGCCAUGGUCG

Supplementary Table S3. **Clinical characteristics of HCC samples.**

No.	Age	Sex	Organ	Pathology diagnosis
01	50	M	Liver	hepatocellular carcinoma
02	57	M	Liver	hepatocellular carcinoma
03	61	F	Liver	hepatocellular carcinoma
04	51	F	Liver	hepatocellular carcinoma
05	56	M	Liver	hepatocellular carcinoma
06	60	M	Liver	hepatocellular carcinoma
07	54	M	Liver	hepatocellular carcinoma
08	36	M	Liver	hepatocellular carcinoma
09	62	F	Liver	hepatocellular carcinoma
10	57	M	Liver	hepatocellular carcinoma
11	45	M	Liver	hepatocellular carcinoma
12	54	M	Liver	hepatocellular carcinoma
13	68	M	Liver	hepatocellular carcinoma
14	52	M	Liver	hepatocellular carcinoma
15	68	F	Liver	hepatocellular carcinoma
16	59	M	Liver	hepatocellular carcinoma
17	63	M	Liver	hepatocellular carcinoma
18	61	M	Liver	hepatocellular carcinoma
19	56	F	Liver	hepatocellular carcinoma
20	35	F	Liver	hepatocellular carcinoma
21	40	M	Liver	hepatocellular carcinoma
22	75	M	Liver	hepatocellular carcinoma
23	53	M	Liver	hepatocellular carcinoma
24	60	F	Liver	hepatocellular carcinoma
25	26	M	Liver	hepatocellular carcinoma
26	51	M	Liver	hepatocellular carcinoma
27	72	F	Liver	hepatocellular carcinoma
28	43	M	Liver	hepatocellular carcinoma
29	26	M	Liver	hepatocellular carcinoma
30	56	M	Liver	hepatocellular carcinoma
31	60	M	Liver	hepatocellular carcinoma
32	65	M	Liver	hepatocellular carcinoma
33	43	M	Liver	hepatocellular carcinoma
34	41	M	Liver	hepatocellular carcinoma
35	45	M	Liver	hepatocellular carcinoma

Supplementary Table S4. List of primers used in this paper.

Gene	Primer	Sequence(5′-3′)
**Primers for plasmid construction**		
PRNAT-HM-sh	forward	CGCGGATCCAGCTTGTGGCCAATGGAAC
	reverse	CGGGGTACCGAACAAAGAATAAAGTTAGAAG
pCMV-TTP	forward	CCGGAATTCATGGCCAACCGTTACACCA
	reverse	CCCAAGCTTTCACTCAGAAACAGAGATGCG
pCMV-PTEN	forward	CCGGAATTCATGACAGCCATCATCAAAGA
	reverse	CCCAAGCTTTTTTCATGGTGTTTTATCCCT
**Primers for luciferase reporter gene assays**		
pGL-HMGA2	forward	TGCTCTAGAAGCTTGTGGCCAATGGAAC
	reverse	GGGGGCCGGCCGAACAAAGAATAAAGTTAGAAG
pGL-PTEN-1702	forward	TGCTCTAGAACTGCATACGATTTTAAGCG
	reverse	GGGGGCCGGCCAATGAAACTGACAAGGTATGGA
pGL-PTEN-3062	forward	TGCTCTAGACAAAGTGCCTCGTTTACCT
	reverse	GGGGGCCGGCCCAAACACAATAACAAATGGACA
pGL-PTEN-1702-mut	forward	GTTTCTTAATGGATATTTTAACCATTTTCAATAAC
	reverse	GTTATTGAAAATGGTTAAAATATCCATTAAGAAAC
**Primers for HMGA2-sh-3p20 Detection**		
HMGA2-sh-3p20	forward	AAATTTTATAGGACTGTTCT
HMGA2-sh-3p21	forward	AATTTTATAGGACTGTTCTT
HMGA2-sh-3p22	forward	ATTTTATAGGACTGTTCTTT
HMGA2-sh-3p23	forward	TTTTATAGGACTGTTCTTTG
HMGA2-sh-5P23	forward	TAAGAACATCATAAAATTTT
HMGA2-sh-5p22	forward	GTAAGAACATCATAAAATTT
HMGA2-sh-5P21	forward	AGTAAGAACATCATAAAATT
HMGA2-sh-5P20	forward	CAGTAAGAACATCATAAAAT
qRT-PCR	reverse	GCGAGCACAGAATTAATACGAC
U6	forward	AGAGCCTGTGGTGTCCG
	reverse	CATCTTCAAAGCACTTCCCT
**Primers for RT-qPCR**		
HMGA2	forward	TGGGAGGAGCGAAATCTAA
	reverse	GGTGAACTCAAGCCGAAG
TTP	forward	CTGACTGCCATCTACGAGAG
	reverse	GAGTTCCGTCTTGTATTTGGG
PTEN	forward	GTCACTGCTTGTTGTTTGC
	reverse	TTCTTTGTTGATAGCCTCCAC
GAPDH	forward	AACGGATTTGGTCGTATTG
	reverse	GGAAGATGGTGATGGGATT
**Primers for RIP-qPCR**		
HMGA2	forward	TGGGAGGAGCGAAATCTAA
	reverse	GGTGAACTCAAGCCGAAG

Primers for miRNAs		
miR-410	forward	AATATAACACAGATGGCCTGT
miR-590	forward	TAATTTTATGTATAAGCTAGT
miR-150	forward	TCTCCCAACCCTTGTACCAGTG
miR-132	forward	TAACAGTCTACAGCCATGGTCG
miR-212	forward	TAACAGTCTCCAGTCACGGCC
miR-145	forward	GTCCAGTTTTCCCAGGAATCCCT
miR-186	forward	CAAAGAATTCTCCTTTTGGGCT
qRT-PCR	reverse	GCGAGCACAGAATTAATACGAC

Supplementary Table S5. List of antibodies used in this paper.

**Gene**	**Manufacture**	**Catalog Number**
HMGA2 Polyclonal antibody	Genetex	GTX100519
Drosha Polyclonal antibody	Proteintech	55001-1-AP
DGCR8 Monoclonal antibody	Proteintech	60084-1-Ig
Dicer Polyclonal antibody	Proteintech	20567-1-AP
TTP Polyclonal antibody	Proteintech	12737-1-AP
PTEN Monoclonal Antibody	Proteintech	60300-1-Ig
β-actin Monoclonal Antibody	Sigma-Aldrich	A2228
Ki67 Monoclonal antibody	Thermo	RM-9106-R7
